# All-trans-retinoic acid activates the pro-invasive Src-YAP-Interleukin 6 axis in triple-negative MDA-MB-231 breast cancer cells while cerivastatin reverses this action

**DOI:** 10.1038/s41598-018-25526-1

**Published:** 2018-05-04

**Authors:** Belén Mezquita, Pau Mezquita, Montserrat Pau, Laura Gasa, Lourdes Navarro, Mireia Samitier, Miquel Pons, Cristóbal Mezquita

**Affiliations:** 1 Departament de Biomedicina, Laboratori de Genètica Molecular, Facultat de Medicina, Universitat de Barcelona, Barcelona, Spain; 2 Institut d’Investigacions Biomèdiques August Pi i Sunyer (IDIBAPS), Barcelona, Spain; 3 Departament de Ciències Bàsiques, Universitat Internacional de Catalunya, Barcelona, Spain; 4 Departament de Química Inorgànica i Orgànica, Secció de Química Orgànica, Laboratori de RMN de biomolècules, Universitat de Barcelona, Barcelona, Spain

## Abstract

All-trans-retinoic acid (RA), the active metabolite of vitamin A, can reduce the malignant phenotype in some types of cancer and paradoxically also can promote cancer growth and invasion in others. For instance, it has been reported that RA induces tumor suppression in tumor xenografts of MDA-MB-468 breast cancer cells while increasing tumor growth and metastases in xenografts of MDA-MB-231 breast cancer cells. The signaling pathways involved in the pro-invasive action of retinoic acid remain mostly unknown. We show here that RA activates the pro-invasive axis Src-YAP-Interleukin 6 (Src-YAP-IL6) in triple negative MDA-MB-231 breast cancer cells, yielding to increased invasion of these cells. On the contrary, RA inhibits the Src-YAP-IL6 axis of triple-negative MDA-MB-468 cells, which results in decreased invasion phenotype. In both types of cells, inhibition of the Src-YAP-IL6 axis by the Src inhibitor PP2 drastically reduces migration and invasion. Src inhibition also downregulates the expression of a pro-invasive isoform of VEGFR1 in MDA-MB-231 breast cancer cells. Furthermore, interference of YAP nuclear translocation using the statin cerivastatin reverses the upregulation of Interleukin 6 (IL-6) and the pro-invasive effect of RA on MDA-MB-231 breast cancer cells and also decreases invasion and viability of MDA-MB-468 breast cancer cells. These results altogether suggest that RA induces pro-invasive or anti-invasive actions in two triple-negative breast cancer cell lines due to its ability to activate or inhibit the Src-YAP-IL6 axis in different cancer cells. The pro-invasive effect of RA can be reversed by the statin cerivastatin.

## Introduction

Triple-negative breast cancers (TNBC) represent 10–17% of all breast cancers and are associated with increased risk of metastasis^1^. Effective treatment for metastatic TNBC is not yet available^2,3^. All-*trans*-retinoic acid (RA), the active metabolite of vitamin A, which is considered an anti-cancer agent, also can promote cancer growth and invasion^4–7^. It has been reported that in tumor xenografts of MDA-MB-231 breast cancer cells RA increases growth and metastasis^7^. Also, *in vivo* experiments show that an RA-enriched diet promotes tumor growth and invasion of T47D403 breast cancer cells and *in vitro* treatment with supraphysiological doses of exogenous RA (10^−6^ M) significantly enhances T47D403 invasion^4^. However, RA acts as a tumor-suppressor in xenografts of MDA-MB-468 breast cancer cells^7^.

The signaling pathways involved in the pro-invasive action of retinoic acid in MDA-MB-231 cells have not been identified. The Src-YAP-IL6 axis controls invasion, metastasis, resistance to therapy, and stemness of MDA-MB-231 breast cancer cells^8,9^. An autoregulatory Src-YAP-IL6-Src loop also operates in colon cancer^10,11^. IL-6 is the first universal transcriptional target of YAP involved in promoting stemness conserved from flies to humans^9,12^. Overexpression of IL-6 induces cancer cell proliferation, angiogenesis, and metastasis through stimulating STAT3, MAPK, and Akt signaling pathways^13^. IL-6 regulates cancer stem cell, mesenchymal stem cell formation, epithelial to mesenchymal transition in cancer and is a contributing factor for chemoresistance^13^.

We show here that RA activates the pro-invasive Src-YAP-IL6 axis in MDA-MB-231 breast cancer cells but inhibits the same axis, migration, and invasion in MDA-MB-468 breast cancer cells. Migration and invasion decreased drastically in both types of cells after interference of the Src-YAP-IL6 axis by the Src inhibitor PP2.

Recently, it has been reported that statins oppose YAP nuclear localization and transcriptional responses in MDA-MB-231 and other breast cancer cells^14^. We show here that cerivastatin can reverse the effect of RA in MDA-MB-231 breast cancer cells by decreasing nuclear PY-YAP localization, IL-6 expression, and the invasive phenotype of these cells. Cerivastatin also decreased cell invasion and viability of MDA-MB-468 breast cancer cells.

## Results

### RA activated the Src-YAP-IL6 axis in MDA-MB-231 breast cancer cells but inhibited the axis in MDA-MB-468 breast cancer cells

**Figure 1:**
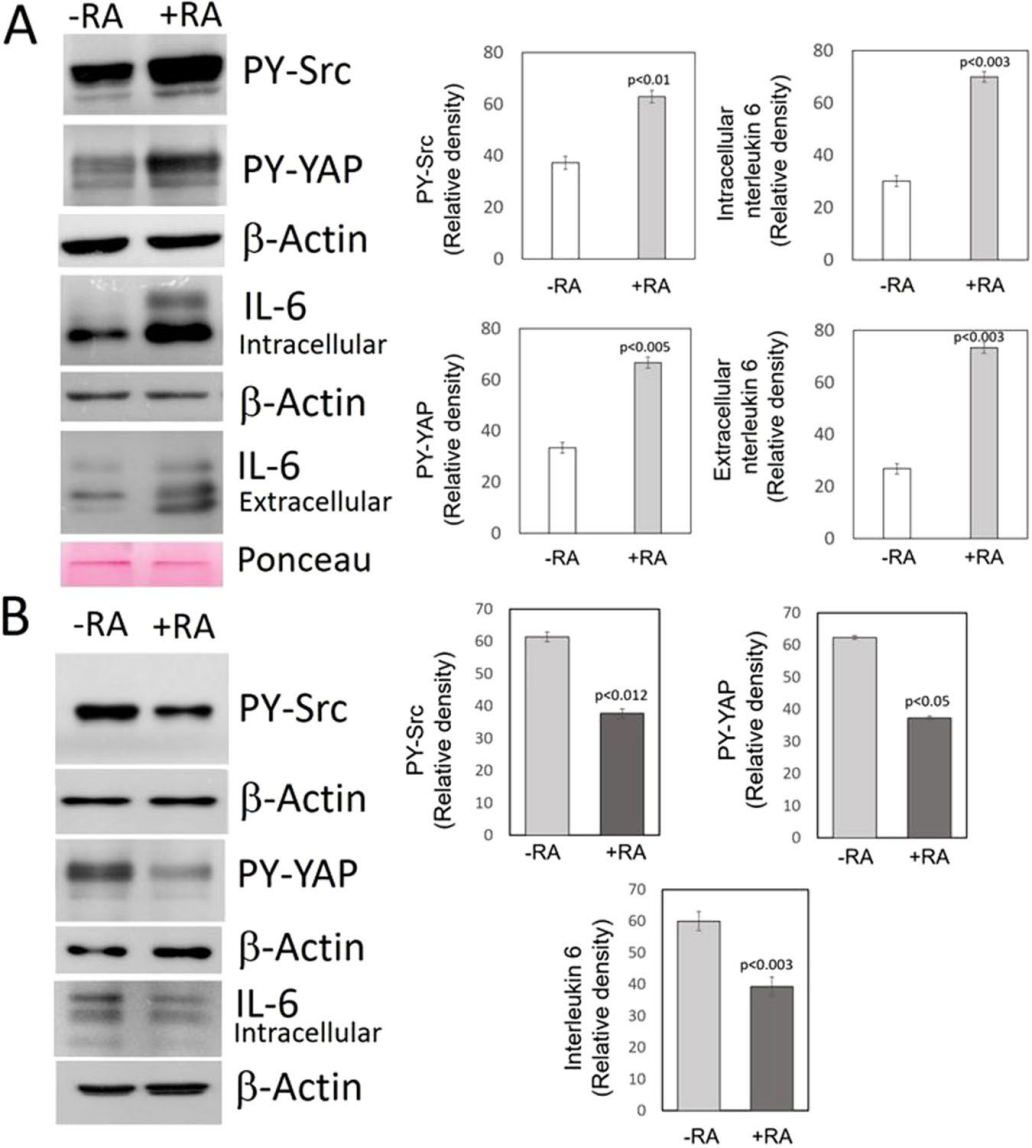
Effect of RA on the Src-YAP-IL6 axis in triple-negative MDA-MB-231 and MDA-MB-468 breast cancer cells. MDA-MB-231 and MDA-MB-468 breast cancer cells were incubated for two days in the absence (-RA) or presence (+RA) of retinoic acid (5 μM). (**A**) Western blots of MDA-MB-231 cells show the increase in tyrosine phosphorylation of Src and YAP determined in nuclear extracts and the increase of IL-6 expression assessed in cell lysates and the culture medium. The bar graphs show quantification of data from three independent experiments. β-Actin and Ponceau staining were used as loading controls. (**B**) Western blots of MDA-MB-468 breast cancer cells show the decrease in tyrosine phosphorylation of Src and YAP determined in nuclear extracts and the decrease of IL-6 expression assessed in cell lysates. The bar graphs show quantification of data from three independent experiments. β-Actin was used as loading control. Full-length figures of the cropped blots are in Supplementary Figures S1–S4.

The Src-YAP-IL6 axis has been identified as a potent inductor of stemness and invasiveness in triple-negative MDA-MB-231 breast cancer cells^9^. In these cells we detected nuclear Src activity, assessed by phosphorylation at tyrosine 418, nuclear PY-YAP (Y357) and IL-6 expression (Fig. 1A).

When MDA-MB-231 breast cancer cells were incubated in the presence of exogenous RA (5 μM) nuclear Src activity increased, and the Src-YAP-IL6 axis was markedly activated. RA increased nuclear PY-YAP as well as the levels of both intracellular and extracellular IL-6 (Fig. 1A).

RA has been previously shown to be tumor suppressive in xenografts of MDA-MB-468 breast cancer cells^7^. Thus, we tested the effect of RA on the Src-YAP-IL6 axis in these cells. In MDA-MB-468 breast cancer cells, the presence of exogenous RA (5 μM) for 48 h decreased nuclear Src activity, nuclear PY-YAP and downregulated IL-6 (Fig. 1B). The observed opposite effects of RA in xenografts of MDA-MB-231 and MDA-MB-468 breast cancer cells^7^ could be ascribed to the different actions of RA on the Src-YAP-IL6 axis we have observed in these cell lines.

**Figure 2:**
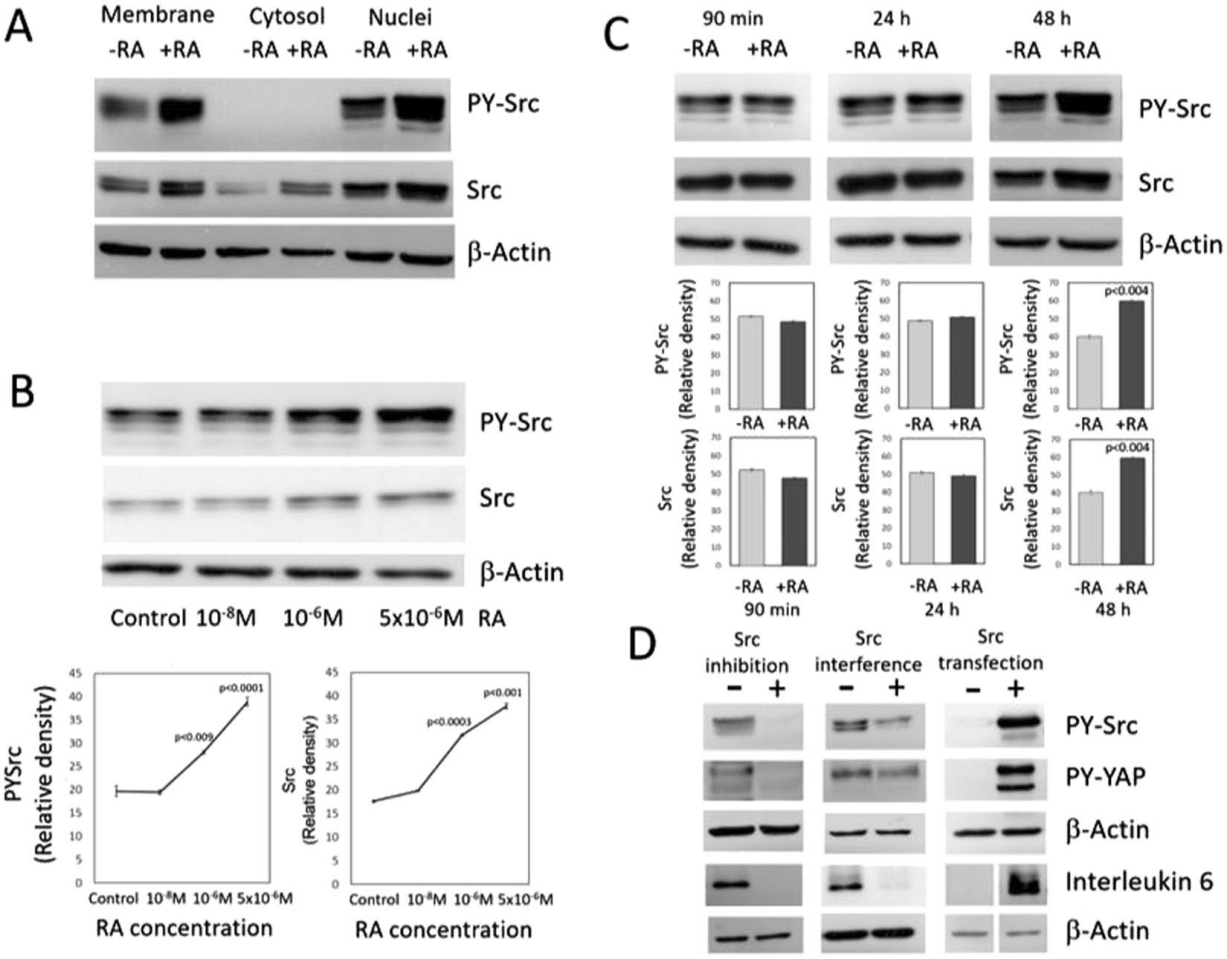
Effect of RA on Src activity and Src expression and consequences of Src inhibition, Src interference, and Src transfection on the Src-YAP-IL6 axis. (**A**) Src activity and Src expression assessed in different cell compartments of MDA-MB-231 breast cancer cells incubated for two days in the absence (−RA) or presence (+RA) of retinoic acid (5 μM). Western blots showed the increase in tyrosine phosphorylation of Src bound to the cell membrane and in the nuclear compartment. RA upregulates the expression of Src in the cell membrane, cytosol, and nuclear extracts. β-Actin was used as loading control. (**B**) Src activity and Src expression determined in MDA-MB-231 cells incubated for 48 h with increasing concentrations of RA. Western blot analyses show an increase in tyrosine phosphorylation of Src and upregulation of Src expression at μM concentrations of RA. The graphs show quantification of data from three independent experiments. (**C**) Src activity and Src expression assessed in MDA-MB-231 breast cancer cells incubated for 90 min, 24 h and 48 h in the presence of 5 μM RA. Western blot analyses show an increase in tyrosine phosphorylation of Src and upregulation of Src expression after 48 h of incubation with 5 μM RA. The graphs show quantification from data from three independent experiments. Full-length figures of the cropped blots are in Supplementary Figures S5–S9. (**D**) Effect of Src inhibition, Src interference, and Src transfection into MDA-MB-231 breast cancer cells on the Src-YAP-IL6 axis. To assess the effect of Src inhibition on the Src-YAP-IL6 axis, control MDA-MB-231 breast cancer cells were treated for two days with the Src inhibitor PP2. Western blot analyses show that Src inhibition by PP2 decreases nuclear PY-YAP and downregulates IL-6 expression. To determine the effect of Src interference on the axis, control MDA-MB-231 breast cancer cells were transfected for three days with ON TARGET plus non-targeting pool as a negative control and with ON TARGET plus targeting Src pool. Src interference by siRNA decreases nuclear PY-YAP and downregulates IL-6. To assess the effect of Src transfection into MDA-MB-231 cells control cells were mock-transfected with only lipofectamine or transfected with Src. Transfection of Src increases nuclear PY-YAP and upregulates Interleukin-6 expression. β-Actin was used as loading control. Full-length figures of the cropped blots are in Supplementary Figures S10–S15.

Src-family kinases are found in the plasma membrane and the nuclear compartment^15,16^. RA treatment of MDA-MB-231 breast cancer cells increases phosphorylation of both membrane and nuclear Src and upregulates Src expression in the cell membrane, cytosol and nuclear compartment (Fig. 2A).

Src activation required cell culture with μM concentrations of RA for 48 h (Fig. 2B). No activation was detected for incubation periods of 90 min or 24 h (Fig. 2C). The long incubation times and the μM concentrations of RA required to observe the effect suggest that it is mediated by the expression of RA-dependent genes that are repressed at physiological levels of RA in breast cancer cells but are transcriptionally responsive at supraphysiological concentrations, as previously reported^4^.

### Nuclear YAP phosphorylation in MDA-MB-231 breast cancer cells was dependent on Src activity

Until recently, activation of YAP was believed to solely depend on the inhibition of the Hippo signaling pathway that retains YAP in the cytoplasm^17^. To assess if YAP activation in MDA-MB-231 breast cancer cells depends on Src activity as observed in other cancer cells^10,11,18^, we used Src inhibition by PP2, Src interference by siRNA and transfection of Src into MDA-MB-231 breast cancer cells. Src inhibition by PP2 and Src interference decreased YAP activity and downregulated IL-6 expression, while Src transfection activated YAP and upregulated IL-6 (Fig. 2D).

### Migration and invasion of MDA-MB-231 increased in breast cancer cells upon treatment with RA, while a decrease was observed in MDA-MB-468 breast cancer cells

**Figure 3:**
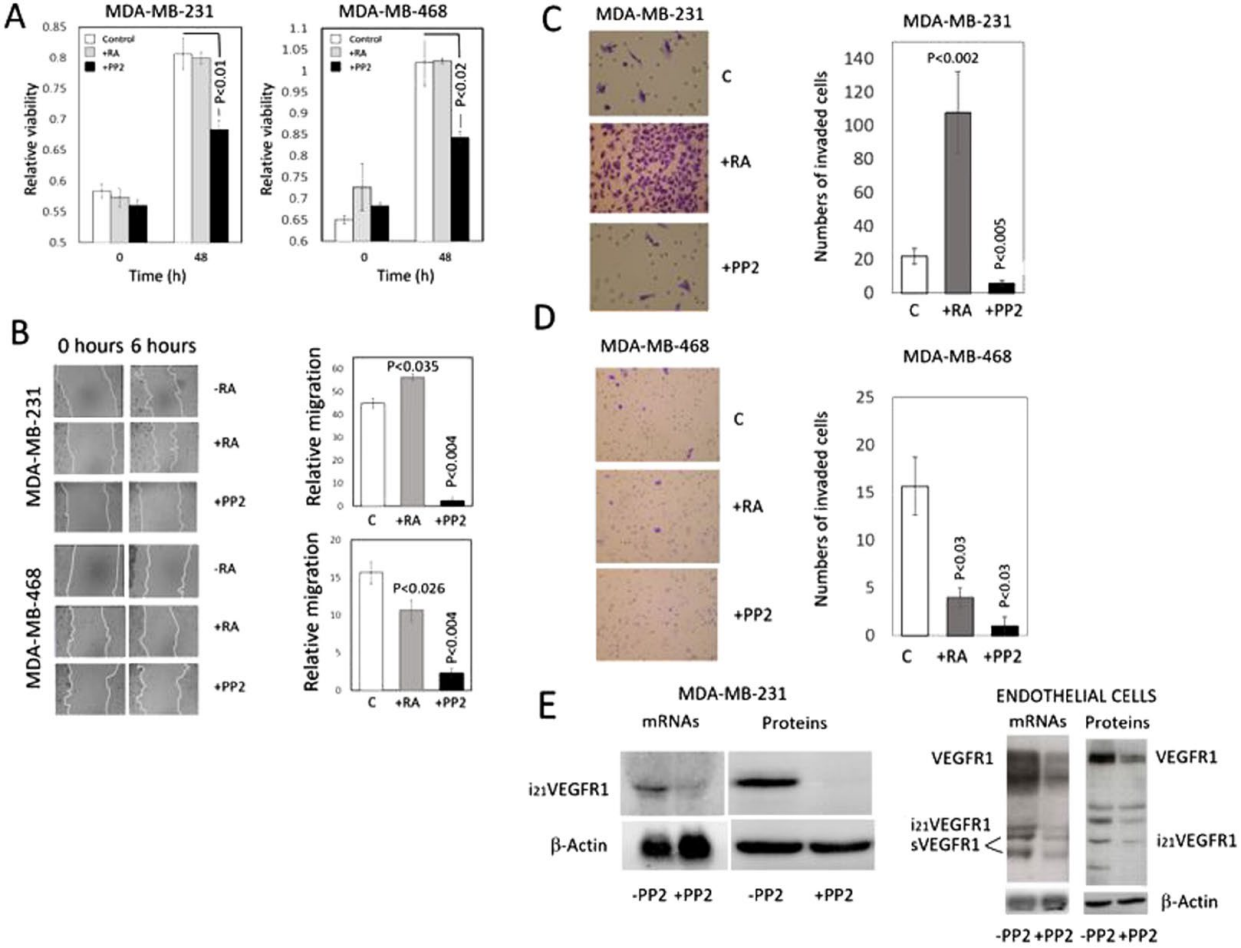
Effect of retinoic acid and the Src inhibitor PP2 on viability, migration, and invasiveness of MDA-MB-231 and MDA-MB-468 breast cancer cells i*n vitro*. Consequences of Src inhibition on the VEGFR1 expression on MDA-MB-231 breast cancer cells and endothelial cells. Prior assessment of viability, migration, and invasion cells were incubated for 48 h in the presence of 5 μM RA or 10 μM PP2. (**A**) Cell viability of MDA-MB-231 and MDA-MB-468 breast cancer cells incubated in the presence of RA or the Src inhibitor PP2. In both MDA-MB-231 and MDA-MB-468 breast cancer cell lines, cell viability did not change in the presence of RA and decreased upon Src inhibition by PP2. The bar graphs show quantification of data from three independent experiments. (**B**) Effect of retinoic acid and Src inhibitor PP2 on the migration of MDA-MB-231 and MDA-MB-468 breast cancer cells i*n vitro*. The cell layer of breast cancer cells treated with either RA or PP2 was streaked with a sterile pipet tip to assess migration, and the wound was allowed to recover for 6 hours. Following recovery, the remaining uncovered wound area was measured, and relative migration was calculated as the percentage of wound area covered by cells. In MDA-MB-231 cells, migration increased by retinoic acid and decreased markedly upon Src inhibition. In MDA-MB-468 cells, migration decreased by retinoic acid and decreased markedly upon Src inhibition. The bar graphs show quantification of data from three independent experiments. (**C** and **D**) Effect of RA and the Src inhibitor PP2 on invasiveness of MDA-MB-231 and MDA-MB-468 cells *in vitro*. Breast cancer cells were treated with either RA or PP2, seeded on polycarbonate filters coated with Matrigel, as described in the Materials and Methods Section, and incubated for 24 h. Quantification of invaded cells represents the mean number of cells per field counting seven random fields at 40× or 20× magnification. Treatment with 5 μM RA markedly increased cell invasion in MDA-MB-231 cells (P < 0.0001 vs. control cells) while a decrease was observed in MDA-MB-468 cells. Treatment with the Src inhibitor PP2 (10μM) suppressed cell invasion in both types of cells. E) Consequences of Src inhibition on the VEGFR1 expression in MDA-MB-231 breast cancer cells and endothelial cells. Cells were incubated in the absence (−) or presence (+) of the Src inhibitor PP2. In MDA-MB-231 breast cancer cells, Northern blot analyses show that the expression of the pro-invasive intracellular isoform i_21_VEGFR1 decreases upon Src inhibition. Western blot analyses show that the protein i_21_VEGFR1 decreases after Src inhibition. In endothelial cells (HUVEC), Northern blot analyses show that the expression of all VEGFR1 isoforms decreases upon Src inhibition. Western blot analyses show that all the isoforms detected by the antibody against the tyrosine domain of VEGFR1 decrease after Src inhibition. β-Actin was used as loading control. Full-length figures of the cropped blots are in Supplementary Figures S16–S19.

It has been previously reported that RA increased tumor growth and metastasis in tumor xenografts of MDA-MB-231 breast cancer cells while becoming suppressive in xenografts of MDA-MB-468 breast cancer cells^7^. Although cell viability of MDA-MB-231 and MDA-MB-468 breast cancer cells did not change *in vitro* upon incubation with RA (Fig. 3A), cell migration and invasion increased markedly in MDA-MB-231 breast cancer cells and decreased in MDA-MB-468 cells (Fig. 3B–D).

### Migration, invasion and cell viability of MDA-MB-231 and MDA-MB-468 breast cancer cells decreased after inhibition of the Src-YAP-IL6 axis by Src inhibitor PP2

Inhibition of the Src-YAP-IL6 axis by Src inhibitor PP2 reduced cell viability, migration, and invasion in both MDA-MB-231 and MDA-MB-468 breast cancer cells (Fig. 3A–D). Also, Src inhibition by PP2 induced downregulation, at transcription and protein levels, of an intracellular isoform of VEGFR1 that initiates transcription in intron 21 (i_21_VEGFR1) (Fig. 3E). In a previous study, we have shown that silencing of i_21_VEGFR1 decreases migration and invasiveness of MDA-MB-231 cells, while transfection of i_21_VEGFR1 increases migration and invasiveness of these cells^19^. No isoforms of VEGFR1, including i_21_VEGFR1, are expressed in MDA-MB-468 breast cancer cells (results not shown). Inhibition of Src by PP2 also decreased the expression of the full-length receptor (VEGFR1), the soluble extracellular isoforms (sVEGFR1) and the truncated intracellular isoforms of VEGFR1 in endothelial cells, both at the transcription and protein levels (Fig. 3E). It has been proposed that VEGFR1 mediates the VEGF-triggered migration of endothelial cells during angiogenesis^20^.

### The statin cerivastatin reverses the action of RA on the Src-YAP-IL6 axis in MDA-MB-231 breast cancer cells and decreases invasion and viability of MDA-MB-231 and MDA-MB-468 breast cancer cells

**Figure 4:**
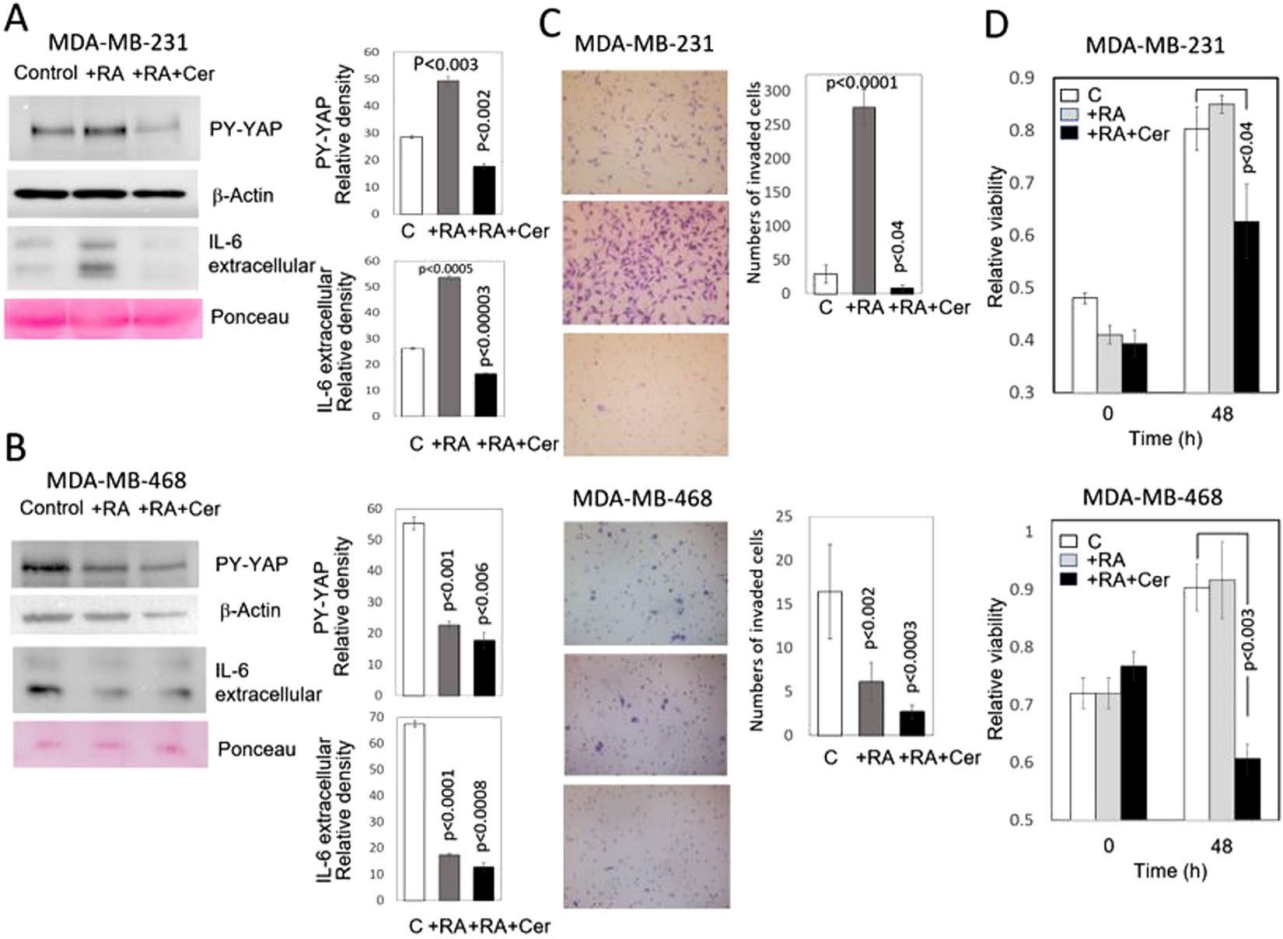
Effect of RA and RA plus cerivastatin on nuclear YAP, IL-6 expression, and invasiveness *in vitro* of triple-negative MDA-MB-231 and MDA-MB-468 breast cancer cells. MDA-MB-231 and MDA-MB-468 breast cancer cells were incubated for two days in the presence (+RA) of retinoic acid (5 μM) or the presence of RA (5 μM) plus cerivastatin (1 μM) (RA + Cer). (**A**) Western blots of nuclear extracts and culture medium of MDA-MB-231 cells treated with RA show the increase of nuclear tyrosine phosphorylated YAP and extracellular IL-6. These actions were reverted when cells were treated with cerivastatin plus retinoic acid. The bar graphs show quantification of data from three independent experiments. β-Actin and Ponceau staining were used as loading control. (**B**) Western blots of nuclear extracts and culture medium of MDA-MB-468 cells treated with RA show the decrease of nuclear PY-YAP and extracellular IL-6. No major additional changes were observed when cells were treated with cerivastatin plus retinoic acid. The bar graphs show quantification of data from three independent experiments. β-Actin and Ponceau staining were used as loading controls. The density of PY-YAP was normalized to the amount of β-Actin. (**C**) To assess the effect of RA and RA + cerivastatin on invasiveness of MDA-MB-231 and MDA-MB-468 cells *in vitro*, treated breast cancer cells were seeded on polycarbonate filters coated with Matrigel, as described in the Materials and Methods Section, and incubated for 24 h. Quantification of invaded cells represents the mean number of cells per field counting seven random fields at 40× or 20× magnification. Treatment with 5 μM RA markedly increased cell invasion in MDA-MB-231 cells (P < 0.0001 vs. control cells) while a decrease was observed in MDA-MB-468 cells. Treatment with RA (5μM) plus cerivastatin (1μM) suppressed cell invasion in both types of cells. (**D**) Cell viability of MDA-MB-231 and MDA-MB-468 breast cancer cells incubated in the presence of RA (5 μM) or RA (5 μM) plus cerivastatin (1 μM). Cells were incubated for 48 h in the presence of RA or RA plus cerivastatin. In both MDA-MB-231 and MDA-MB-468 breast cancer cell lines, cell viability did not change in the presence of RA and decreased upon incubation in the presence of RA plus cerivastatin. The bar graphs show quantification of data from three independent experiments. Full-length figures of the cropped blots are in Supplementary Figures S20–S21.

Since statins oppose YAP nuclear localization and transcriptional responses induced by YAP in MDA-MB-231 and other breast cancer cells^14^, we wanted to assess whether cerivastatin can reverse the action of RA on the Src-YAP-IL6 axis in MDA-MB-231 breast cancer cells and decrease cell invasion and viability. MDA-MB-231 and MDA-MB-468 cells were incubated with exogenous RA (5 μM) for 48 h in the presence or absence of cerivastatin (1 μM). Nuclear PY-YAP and IL-6 decreased in MDA-MB-231 and MDA-MB-468 breast cancer cells incubated in the presence of RA plus cerivastatin (Fig. 4A,B). Cell invasion and viability also decreased in both MDA-MB-231, and MDA-MB-468 incubated with RA plus cerivastatin (Fig. 4C,D).

## Discussion

We report here that treatment of triple-negative breast cancer cells with RA *in vitro* activated the pro-invasive Src-YAP-IL6 axis in MDA-MB-231 cells and inactivated this signaling pathway in MDA-MB-468 cells. This observation is in line with the reported action of RA in xenotransplantation experiments in mice where it increased tumor growth and metastasis in tumor xenografts of MDA-MB-231 cells but decreased the invasive phenotype in MDA-MB-468 breast cancer cells^7^.

Other pro-invasive actions of RA have been reported using *in vivo* experiments with an RA-enriched diet that promoted tumor growth and invasion of T47D403 breast cancer cells, and *in vitro* treatment with supraphysiological doses of exogenous RA (10^−6^ M) that significantly promoted T47D403 breast cancer cell invasion^4^. Promotion of migration and invasion was also reported in neuroblastoma cells treated with retinoic acid^21^. An association of YAP activity and RA signaling with an increase in migration also has been observed in human neural crest cells^22^.

Our observation of pro-invasive actions of RA in MDA-MB-231 breast cancer cells is in apparent contradiction with our previous finding that RA downregulated putative pro-invasive molecules such as i21VEGFR-1 and Notch-3, and upregulated putative anti-invasive molecules as miR-200c^19,23,24^. Despite these actions, we report here that the effect of exogenous RA activating the Src-YAP-IL6 axis in MDA-MB-231 breast cancer cells and therefore increasing their invasion overrides putative anti-invasive mechanisms produced by RA in these cells.

Activation of Src should be relevant for activation of YAP and upregulation of IL-6 in MDA-MB-231 breast cancer cells since inhibition of Src by PP2 or silencing Src expression in these cells decreases PY-YAP and downregulates IL-6 expression (Fig. 2D). On the contrary, transfection of Src increases nuclear PY-YAP and IL-6 expression (Fig. 2D).

The mechanism of Src activation induced by RA is not known at present. Mechanisms independent of transcription have been reported^25^. However, the activation of the Src-YAP-IL6 axis shown here should be the consequence of a genomic action of RA, given the 48 h delay following incubation with supraphysiological concentrations of RA (5 μM). Extragenomic effects of RA in breast cancer cells are produced faster and with lower levels of RA^4^.

Exogenous RA induces differential gene expression in MDA-MB-231 and MDA-MB-468 cells increasing the expression of specific genes such the membrane mucin MUC4 in MDA-MB-231 breast cancer cells but not in MDA-MB-468 cells where the MUC4 gene is hypermethylated^7^. Overexpression of MUC4 in triple-negative breast cancer cells augmented cell migration *in vitro* and metastasis, while its knockdown reduced aggressiveness *in vitro*, and tumorigenesis and metastasis *in vivo*^26^. MUC4 is an attracting candidate for Src activation because cell knockdown of MUC4 in pancreatic carcinoma decreased Src tyrosine phosphorylation significantly^27^.

Our results indicate that Src inhibition by PP2 inactivates the Src-YAP-IL6 axis in triple-negative MDA-MB-231 and MDA-MB-468 breast cancer cells and suppresses cell migration, invasion, and viability of these cells (Fig. 3A–D). Also, we report that Src inhibition downregulates pro-invasive VEGFR1 molecules^19,20,23,24^ in MDA-MB-231 breast cancer cells and endothelial cells (Fig. 3E). Src inhibitors have been suggested as a therapeutic approach for triple-negative breast cancer^28^.

Our observation that the statin cerivastatin can reverse the pro-invasive phenotype induced by RA in MDA-MB-231 breast cancer cells by decreasing nuclear PY-YAP and IL-6 production (Fig. 4) is in line with the action recently reported of statins inhibiting the YAP-dependent transcription of the pro-metastatic gene RHAMM in these cells^29–31^. Although the concentration of statins used to inhibit YAP nuclear translocation (1 μM) largely exceeds the plasma concentrations of statins used for cardiovascular disease prevention (10 to 200 nM)^32^, statins are the object of intensive studies in cancer recurrence and mortality^33,34^. Further work will show whether combined therapy using RA and statins can reverse the malignant phenotype induced by RA in specific breast cancer cells while preserving the anti-tumor effects of retinoids as differentiation enhancers.

## Materials and Methods

### Cell Culture and Reagents

MDA-MB-231 and MDA-MB-468 cells, obtained from the American Type Culture Collection (ATCC) were maintained in Dulbecco’s modified Eagle medium/Ham’s F12 (1:1) supplemented with 10% fetal bovine serum (FBS) and 2 mM glutamine. Human umbilical vein endothelial cells (HUVEC) were obtained from Clonetics (cc-2517) and cultured following the provider’s recommendations and media. Cells were maintained in a 5% CO2 atmosphere at 37 °C. All cell lines were used at low passage (<20) and regularly tested against mycoplasma.

All-*trans*-retinoic acid (RA) (Sigma) was dissolved in ethanol (stock solution 2.5 mM). The reagent was diluted to its final concentration (5 μM) using cell culture medium. The selective Src family-tyrosine kinase inhibitor, 4-amino-5- (4-chlorophenyl)-7-(t-butyl) pyrazolo (3,4-d) pyrimidine (PP2) (Calbiochem, San Diego, CA)^35^ suspended in 1% dimethyl sulfoxide (DMSO) was added to the culture medium at a concentration of 10 μM. Cerivastatin (Sigma) was dissolved to a final concentration of 1 μM in the culture medium. Cells were treated for 48 hrs with either ethanol, DMSO, 10 μM PP2, 1 µM cerivastatin or 1 µM cerivastatin plus RA (5 μM).

### Preparation of RNA, Northern

RNA was obtained using RNeasy Mini kit from Qiagen (Hilden, Germany). Northern blot analysis has been previously described^19^.

### Western Blotting

Cells were lysed on ice in NP40 lysis buffer: 150 mM NaCl, 20 mM HEPES (pH 7.5), 0.5% NP-40, a cocktail of protease inhibitors (Complete, Roche, Basel, Switzerland) and phosphatase inhibitors (Calbiochem, Merck Darmstadt, Germany). Nuclear extracts were obtained using Biovision’s Nuclear/Cytosol Fractionation Kit. Cell and nuclear extracts were separated on SDS-PAGE (10% acrylamide), transferred to PVDF membranes, and probed with antibodies. Antibodies for Src, Phospho-Src (Tyr416), YAP, IL-6, and VEGFR-1 were obtained from Cell Signaling Technology (Beverly, MA). Antibody for phospho-YAP (Y357) was from Abcam. Antibody for β-Actin was from Sigma.

### Transfection of Src

Transfections were carried using Lipofectamine 2000 (Invitrogen) following the manufacturer’s instructions. Src plasmid was from Origene (sc125208).

### Interference of Src

Interference of Src was accomplished using a pre-designed ON-TARGET SMART pool from ThermoScientific (L-003175). ON-TARGET plus non-targeting pool was used as a negative control. Transfections were carried using Lipofectamine 2000 (Invitrogen) following the manufacturer’s instructions. The transfection efficiency, between 80% and 90% was assessed using the BLOCK-iT Fluorescent Oligo (Invitrogen).

### Cell Migration and Invasion Assays

Migration was measured by wound healing assay, in which cells were grown to 80% confluence, streaked with a sterile pipet tip, and the wound area was measured. Cells were allowed to recover for 6 hours, after which the remaining wound area was measured. Cell invasion assays were done using Corning Matrigel Invasion Chamber 24-Well Plate (Corning, Tewksbury, MA) (polycarbonate filter of 8 μm pore size). MDA-MB-231 and MDA-MB-468 cells were trypsinized, and a suspension of 6 × 10^4^ cells in their growing medium was layered in the upper compartment. The lower chamber contained fresh culture medium with 10% FBS as a chemoattractant. After 24 h, the cells on the upper surface of the membrane were removed with a cotton swab, and the invading cells on the underside of the membrane were fixed, stained, and counted in seven random fields at 40 × or 20 × magnification.

### Cell Viability Assay

Cell viability was assessed by using an MTS colorimetric assay (CellTiter 96, AQueous One, Promega, Madison, WI). Cancer cells were seeded at a density of 10^4^ cells/well in 96-well microtiter plates and allowed to attach. After three days without renewal of the culture medium RA or the Src inhibitor PP2 were added to the culture medium and incubated for two days. Subsequently, the CellTiter 96 AQueous One Solution Reagent was added to the cells and allowed to incubate for two hours. Absorbance was read at 490 nm with a 96-well plate reader.

### Statistical Analyses

Data from three independent experiments are expressed as means plus or minus SD. Statistical analyses were performed in Excel (Microsoft, Redmond, WA) using Student t-test. Values were considered significantly different if *p* values were less than 0.05.

### Data Availability

All data generated or analyzed during this study are included in this published article.

## Electronic supplementary material

Supplemenary Information
