# Supplementary material for: All-trans-retinoic acid activates the pro-invasive Src-YAP-Interleukin 6 axis in triple-negative MDA-MB-231 breast cancer cells while cerivastatin reverses this action
Source: Sci Rep. 2018 May 4;8:7047. doi: 10.1038/s41598-018-25526-1 (PMC5935706; doi:10.1038/s41598-018-25526-1)

Supplementary Figures

All-trans-retinoic acid activates the pro-invasive Src-YAP-Interleukin 6 axis in triple-negative MDA-MB-231 breast cancer cells while cerivastatin reverses this action.

Belén Mezquita, [1,2,3] Pau Mezquita,[3] Montserrat Pau, [1] Laura Gasa,[3] Lourdes Navarro,[3] Mireia Samitier,[1] Miquel Pons[4] and Cristóbal Mezquita*[2]

1. Departament de Biomedicina. Laboratori de Genètica Molecular, Facultat de Medicina, Universitat de Barcelona, Barcelona, Spain
2. Institut d’Investigacions Biomèdiques August Pi i Sunyer (IDIBAPS), Barcelona, Spain
3. Departament de Ciències Bàsiques, Universitat Internacional de Catalunya, Barcelona, Spain
4. Departament de Química Inorgànica i Orgànica. Secció de Química Orgànica. Laboratori de RMN de biomolècules. Universitat de Barcelona.

*Correspondence to: Cristóbal Mezquita, Institut d’Investigacions Biomèdiques August Pi i Sunyer (IDIBAPS), Casanova 143, 08036 Barcelona, Spain. email address: cmezquita@ub.edu

Figure S1. Full-length images of the cropped blots presented in the main figure 1 A (PY-Src, PY-YAP and actin).

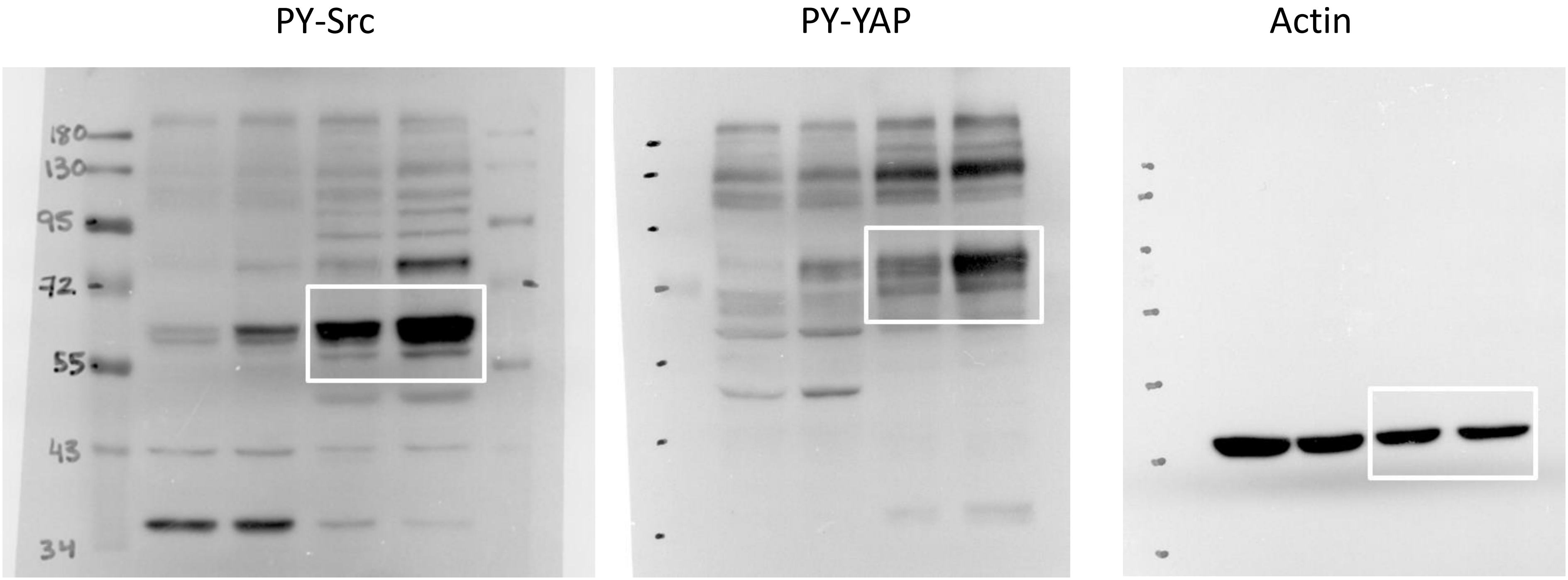

Figure S2. Full-length images of the cropped blots presented in main figure 1 A (IL6, actin and Ponceau)

IL6 Intracellular

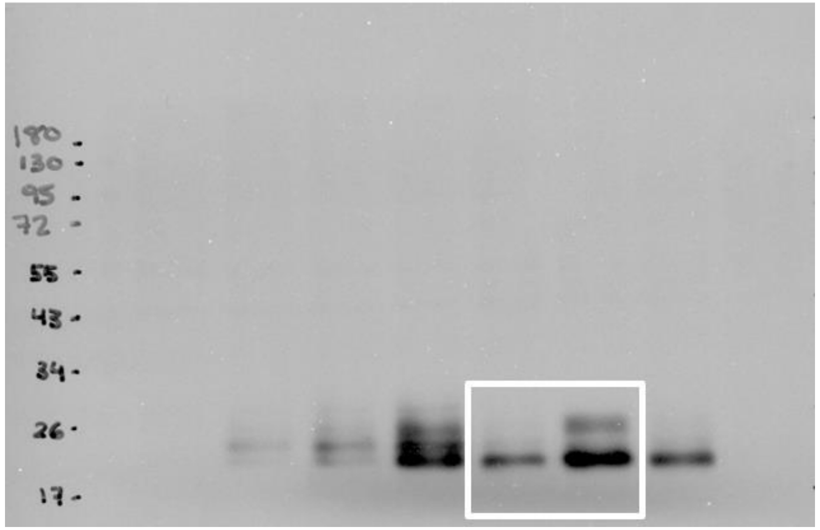

IL6 Extracellular

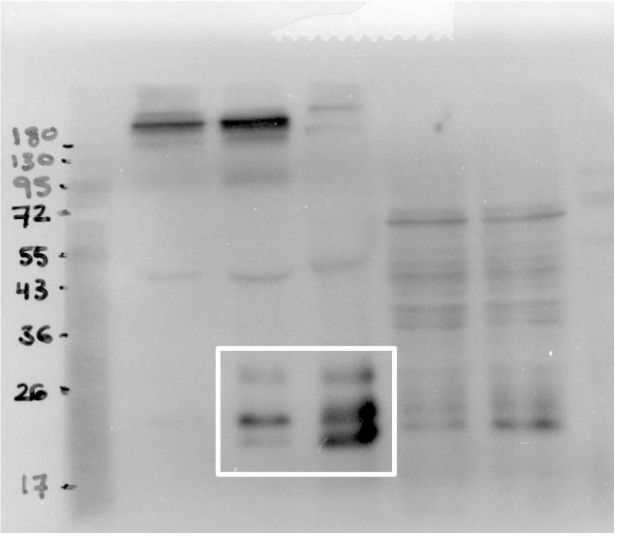

Actin

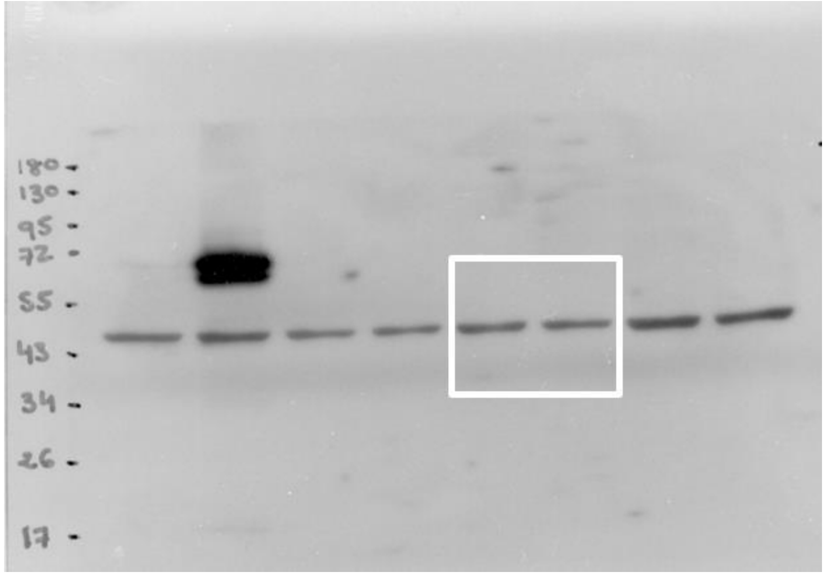

Ponceau

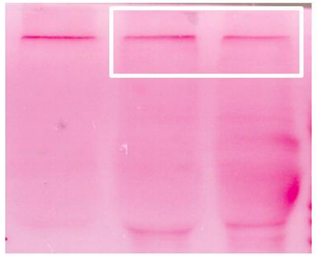

Figure S3. Full-length images of the cropped blots presented in main figure 1 B (PY-Src, PY-YAP and actins)

PY-Src

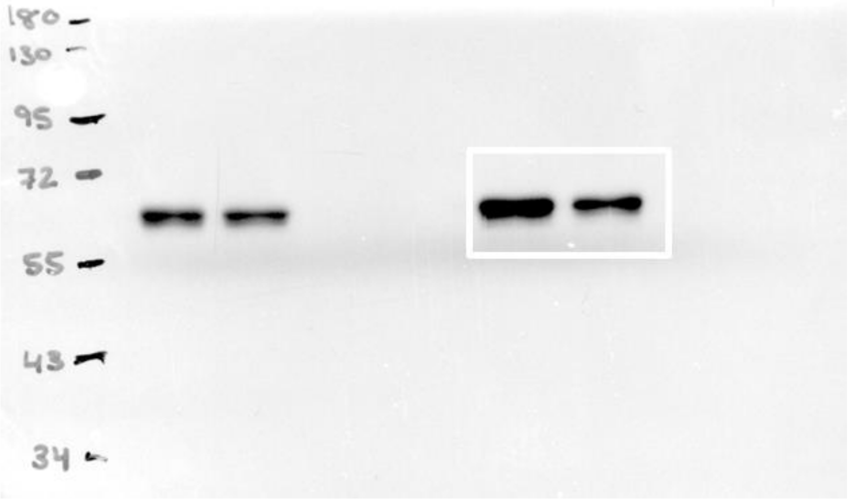

PY-YAP

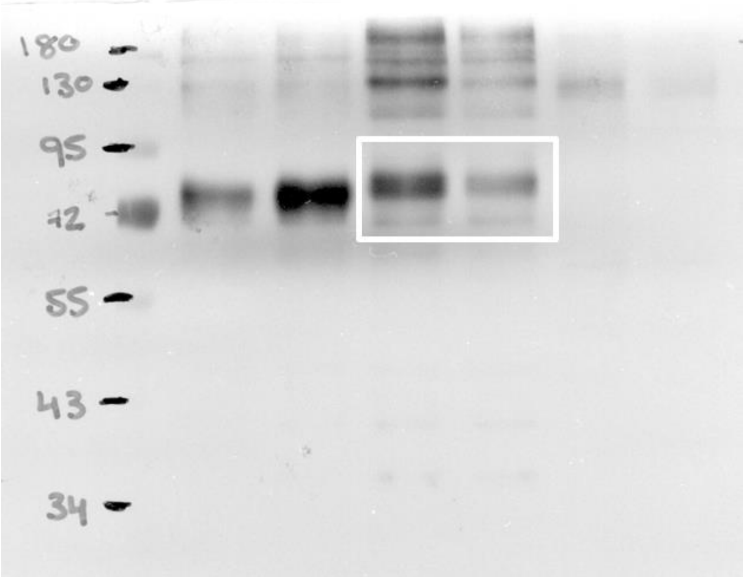

Actin

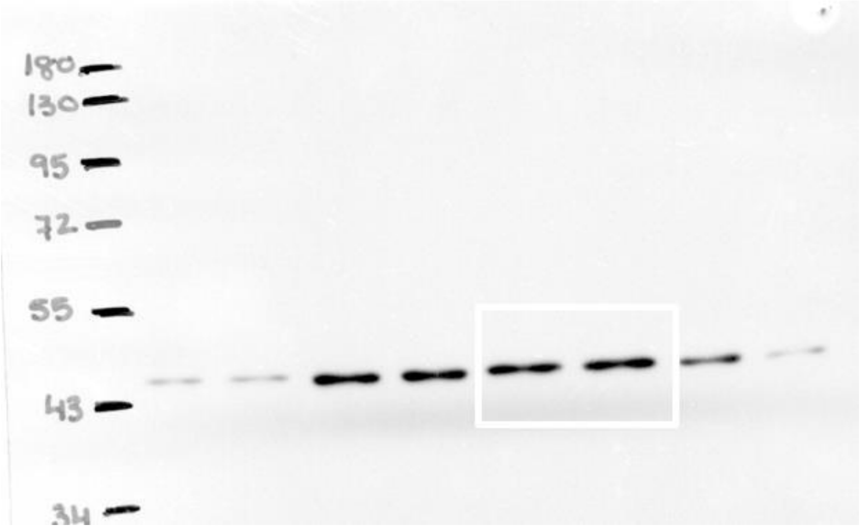

Actin

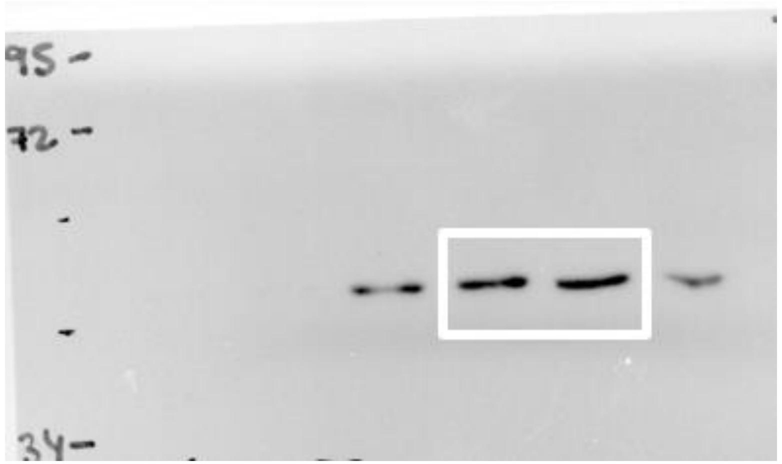

Figure S4. Full-length images of the cropped blots presented in main figure 1 B (IL6 and actin)

IL6 Intracellular

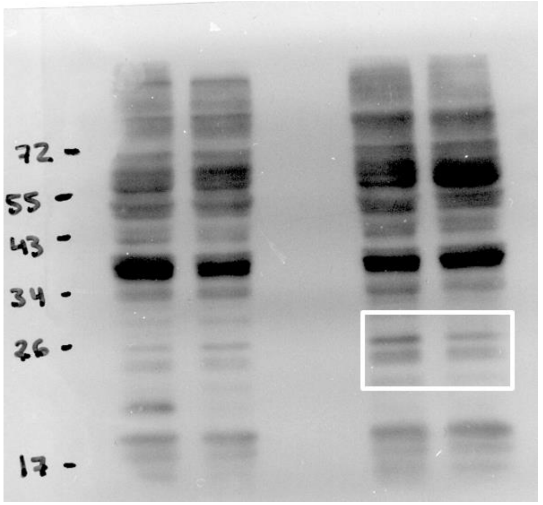

Actin

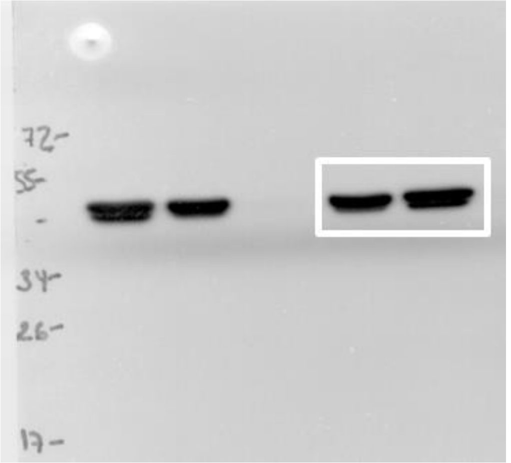

Figure S5. Full-length images of the cropped blots presented in main figure 2A

PY-Src

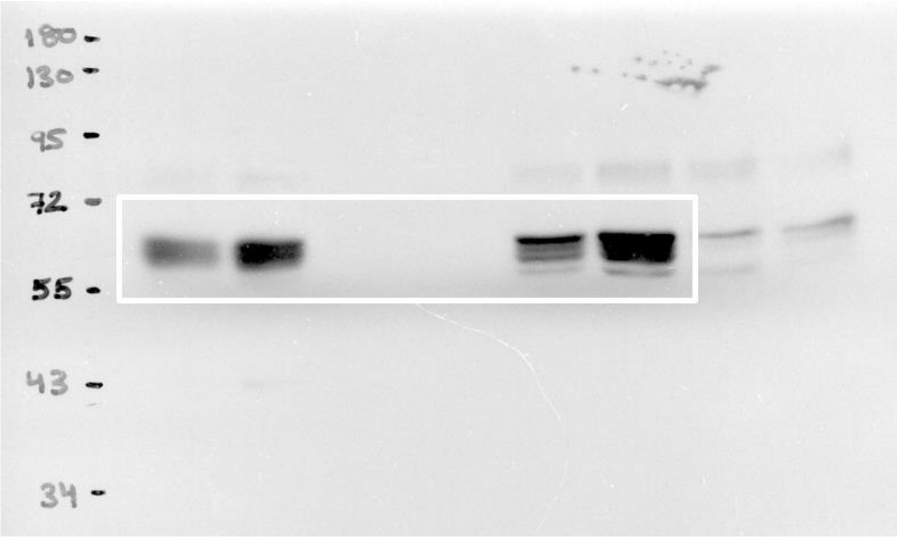

Actin

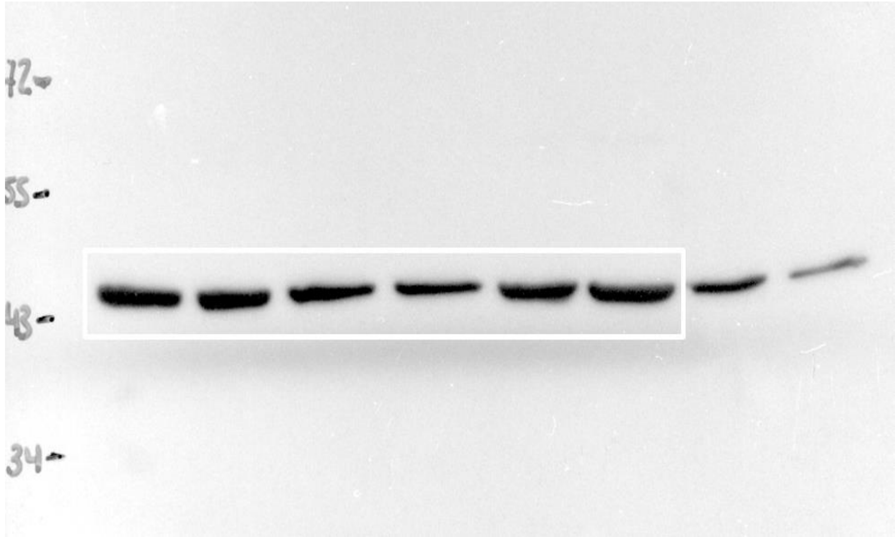

Src

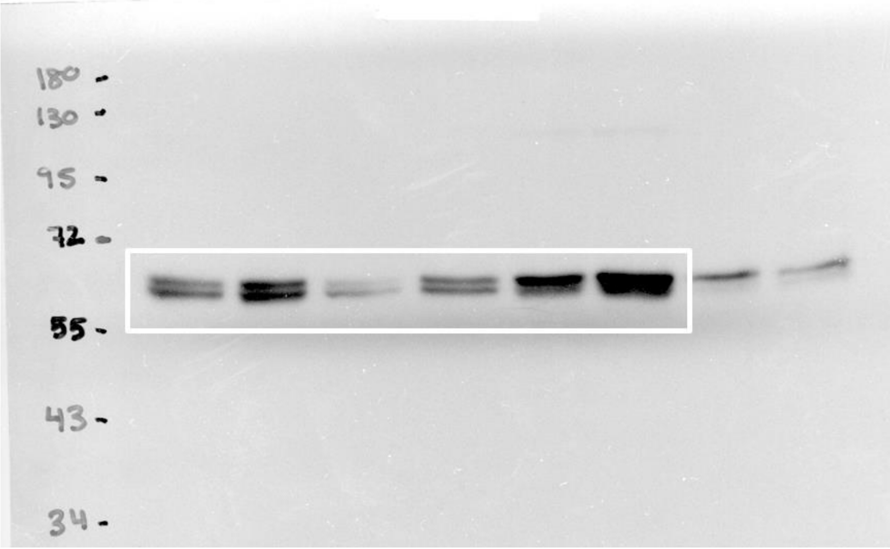

Figure S6. Full-length images of the cropped blots presented in main figure 2B.

PY-Src

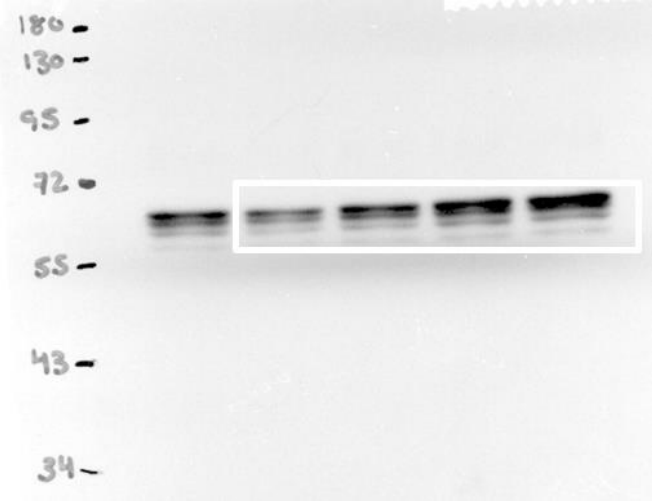

Actin

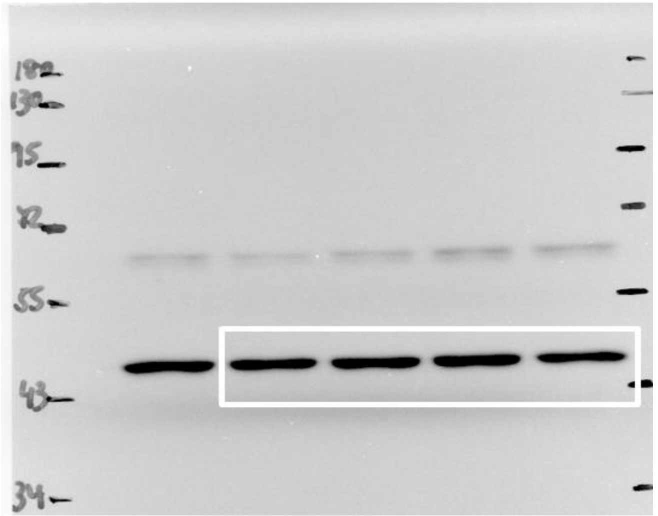

Src

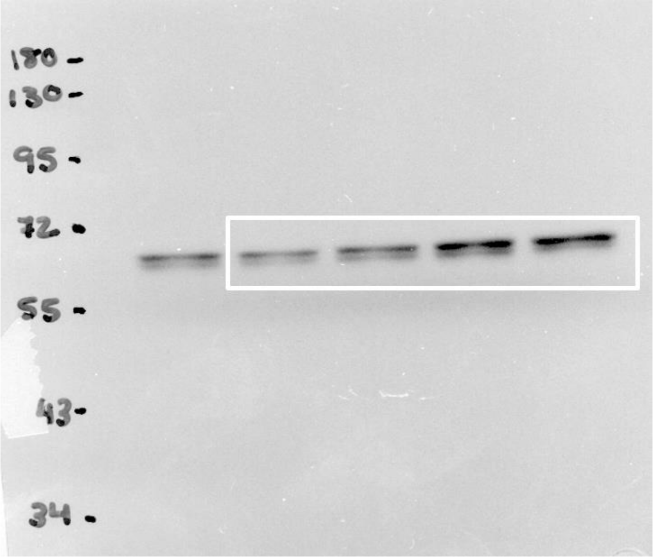

Figure S7. Full-length images of the cropped blots presented in main figure 2C (90 min).

PY-Src

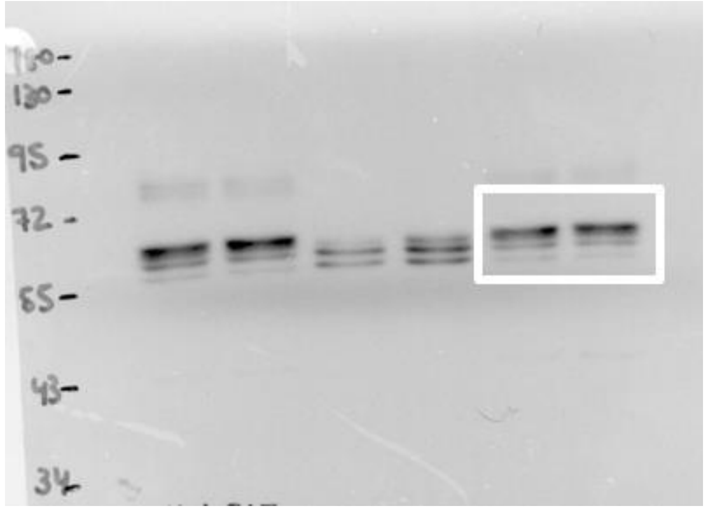

Actin

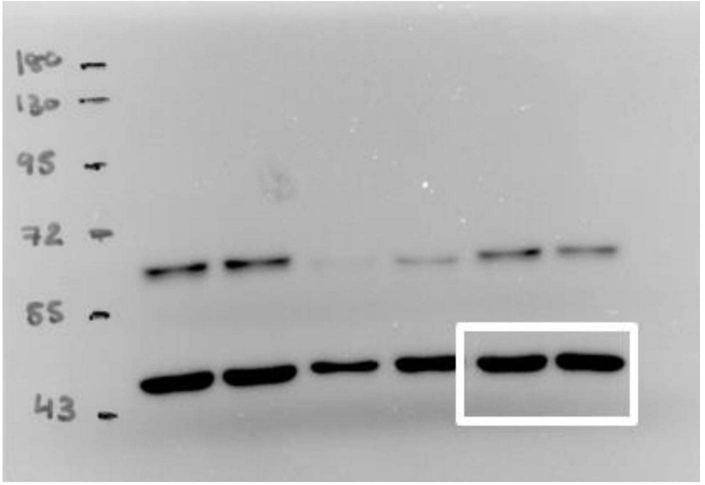

Src

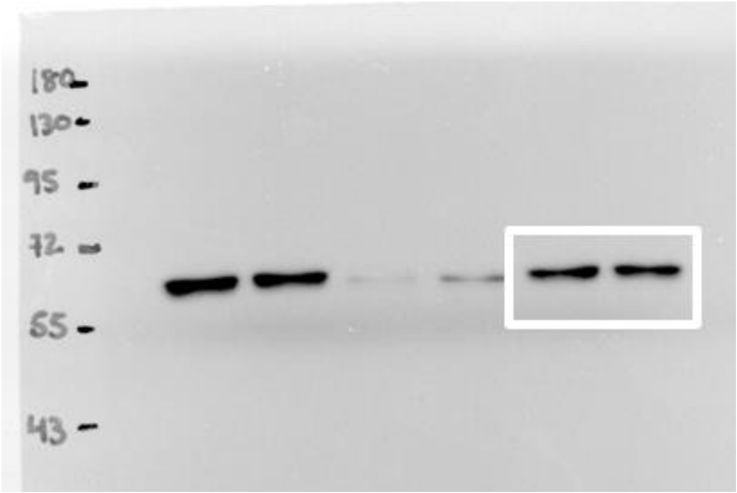

Figure S8. Full-length images of the cropped blots presented in main figure 2C (24 h).

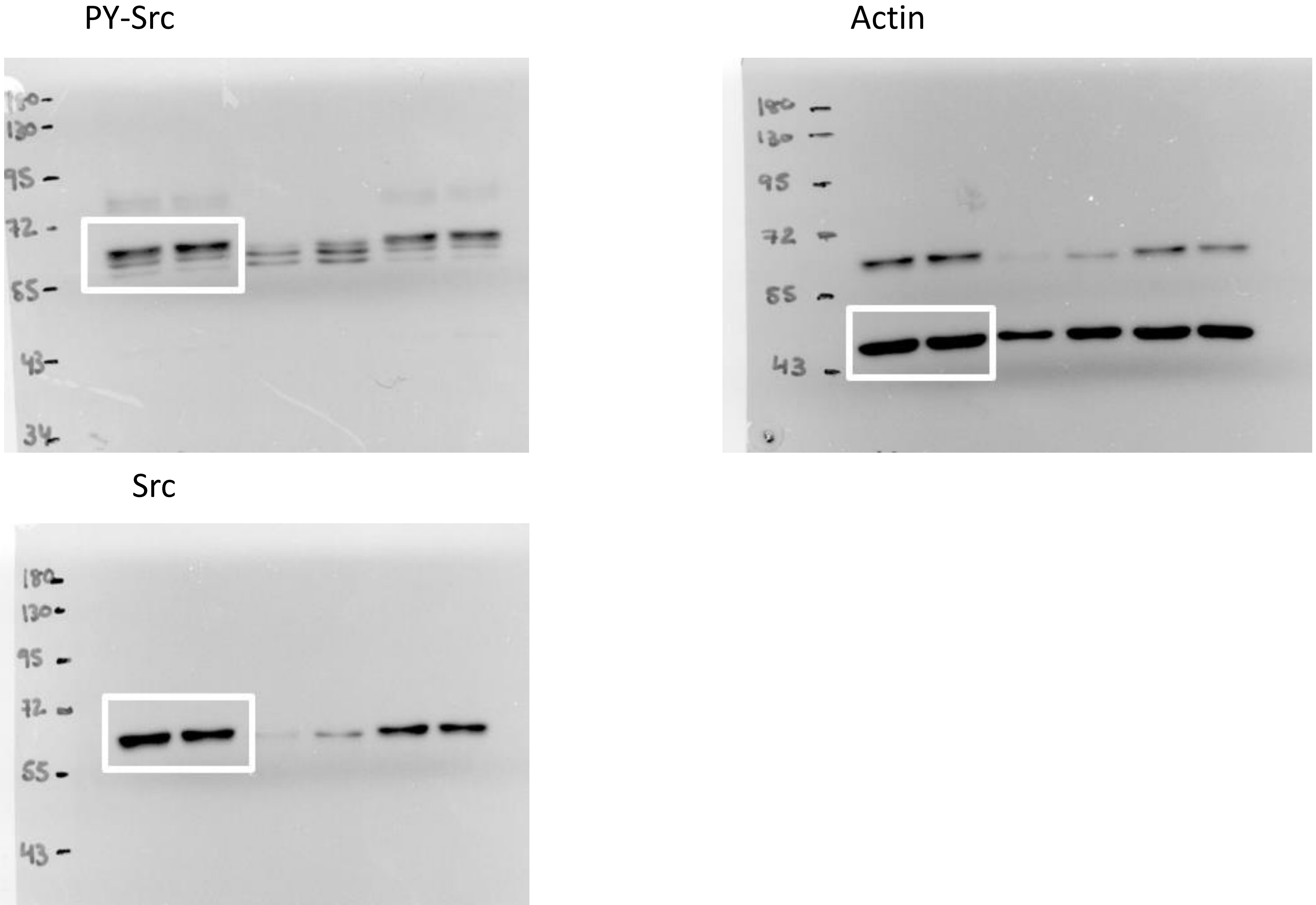

Figure S9. Full-length images of the cropped blots presented in main figure 2C (48 h).

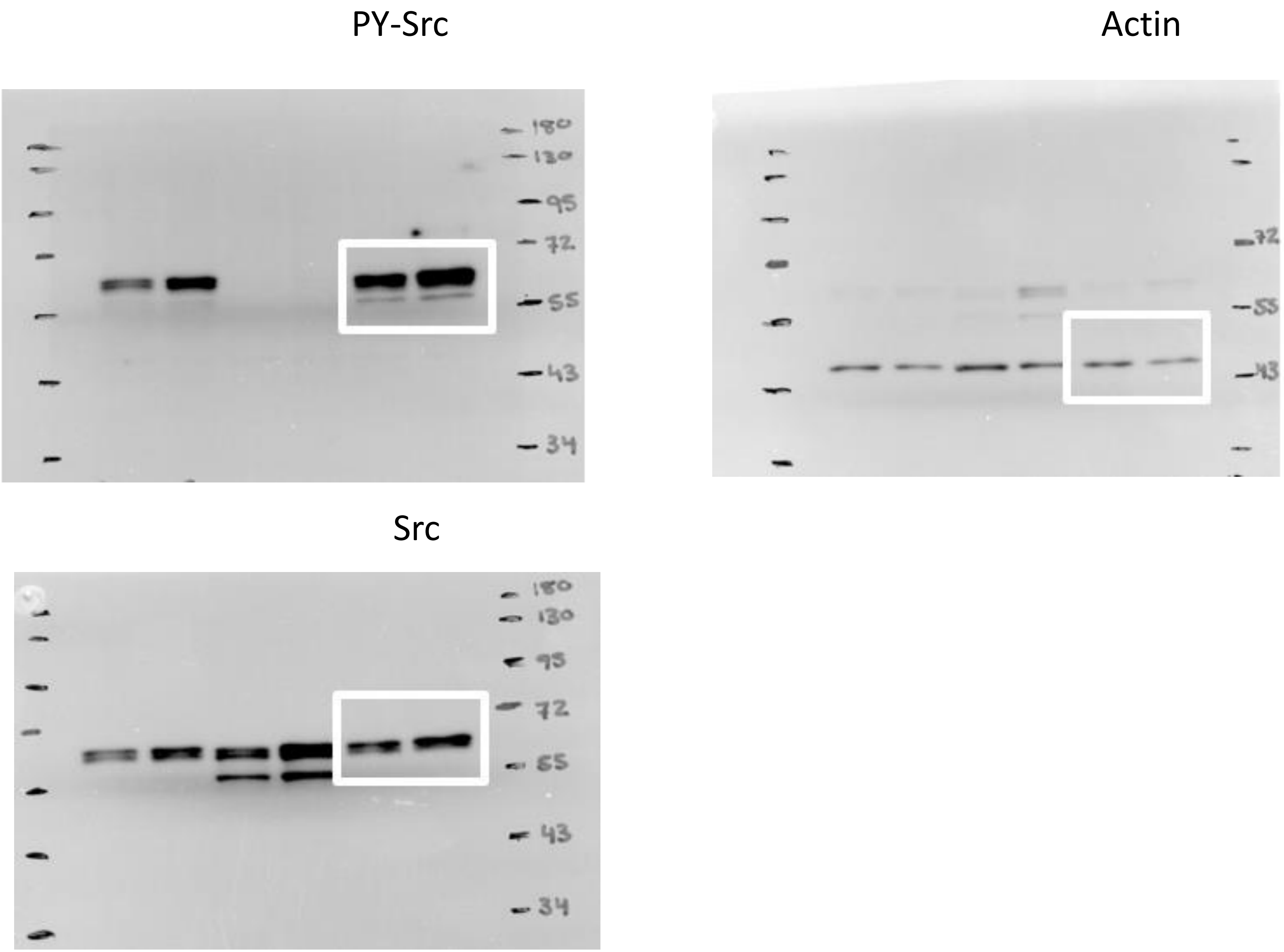

Figure S10. Full-length images of the cropped blots presented in main figure 2D Src inhibition (PY-Src, PY-YAP and actin).

PY-Src

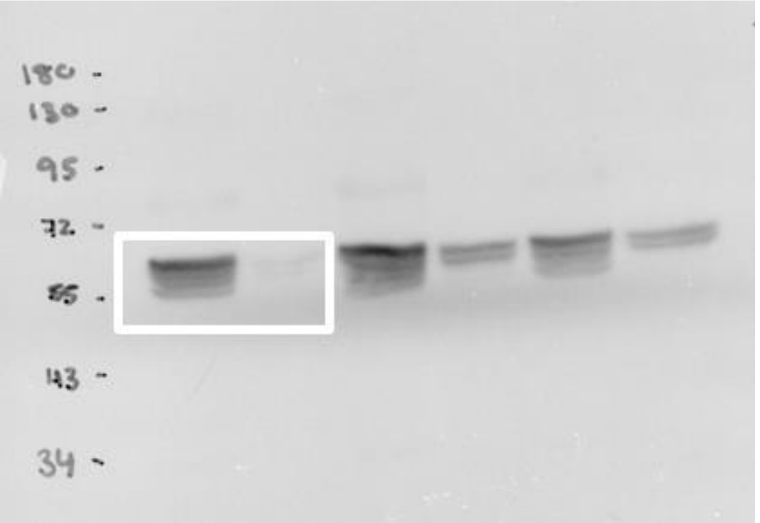

PY-YAP

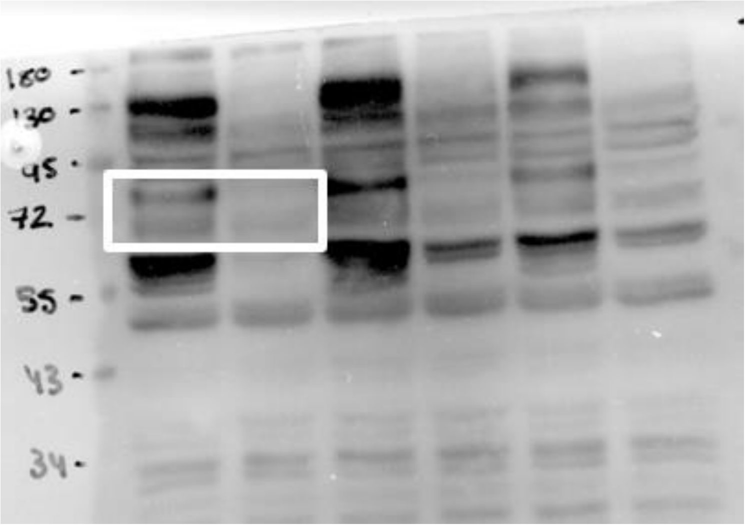

Actin

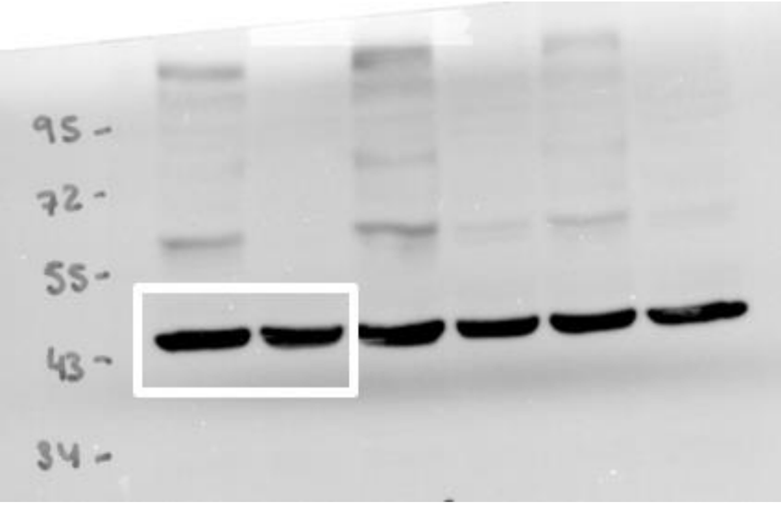

Figure S11. Full-length images of the cropped blots presented in main figure 2D Src inhibition (IL6 and actin).

IL6

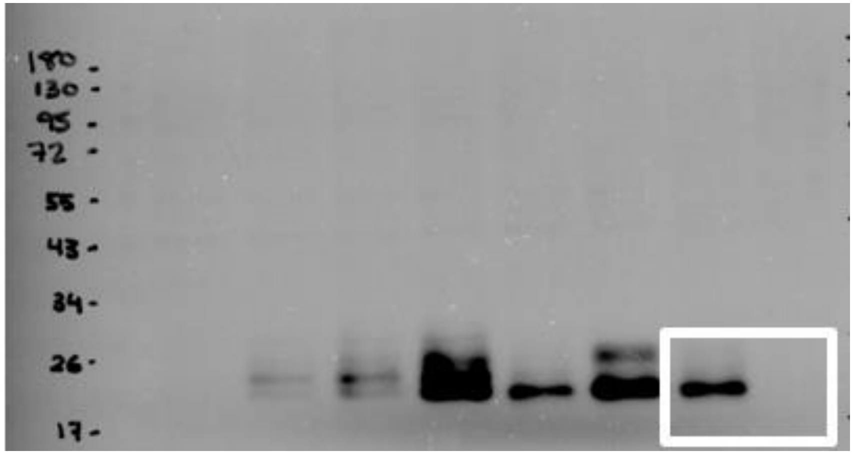

Actin

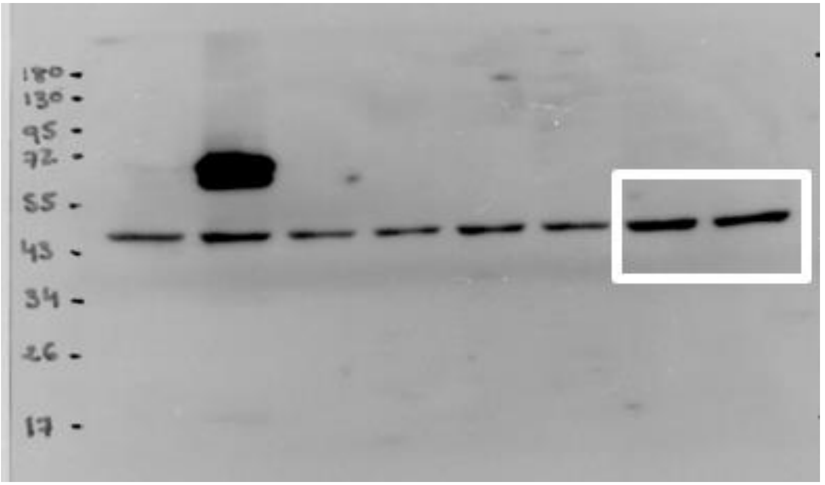

Figure S12. Full-length images of the cropped blots presented in main figure 2D Src interference (PY-Src and PY-YAP and actin).

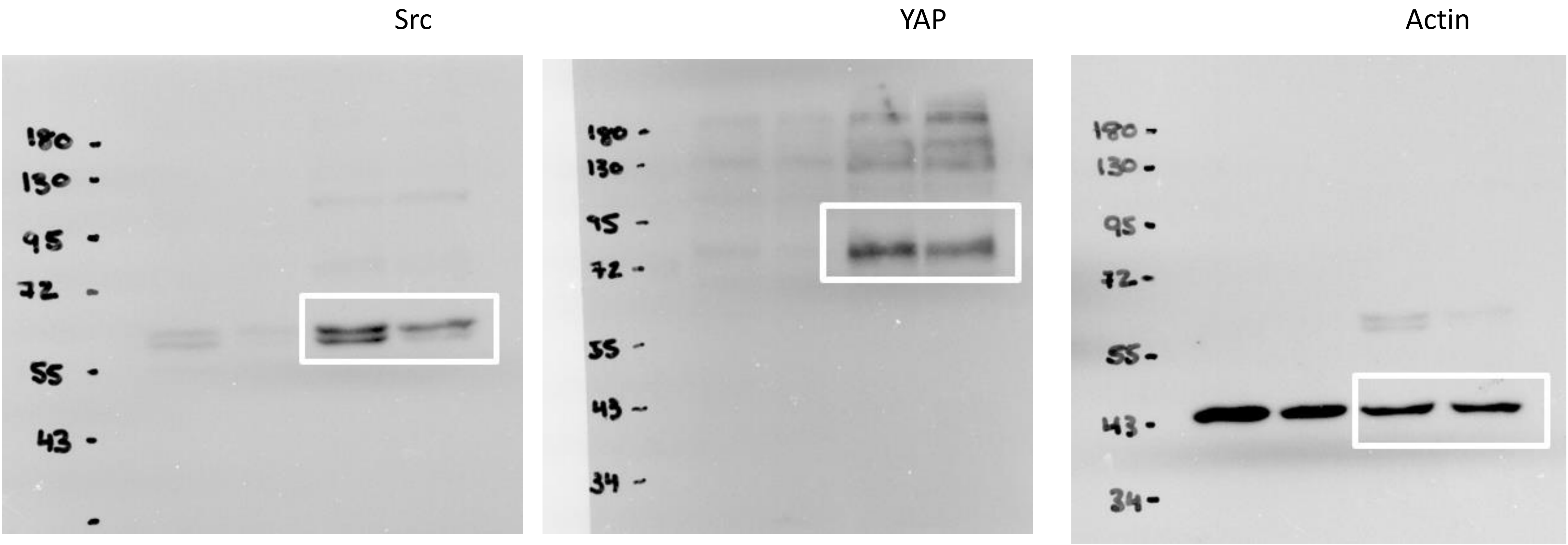

Figure S13. Full-length images of the cropped blots presented in main figure 2D Src interference (IL6 and actin).

IL-6

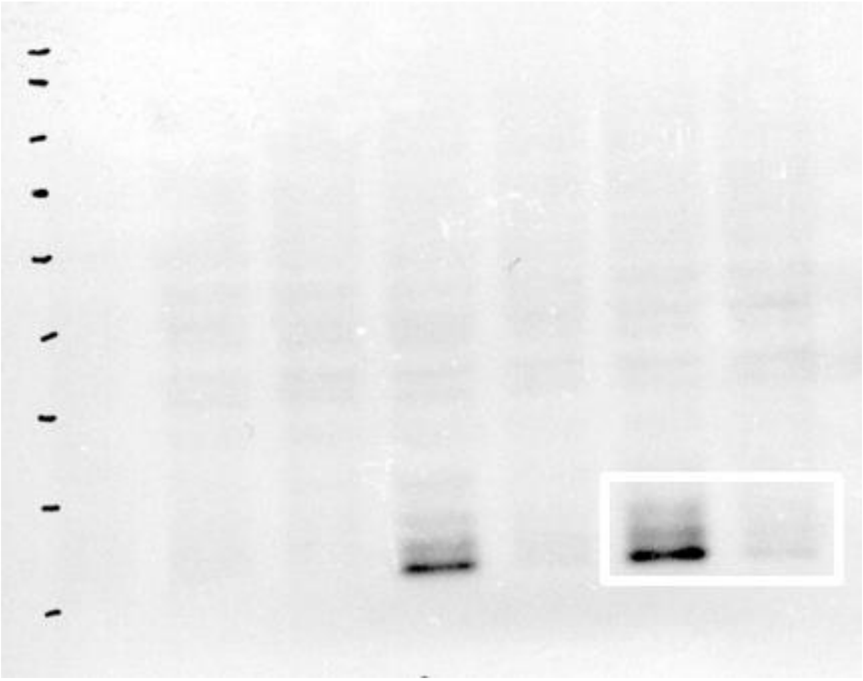

Actin

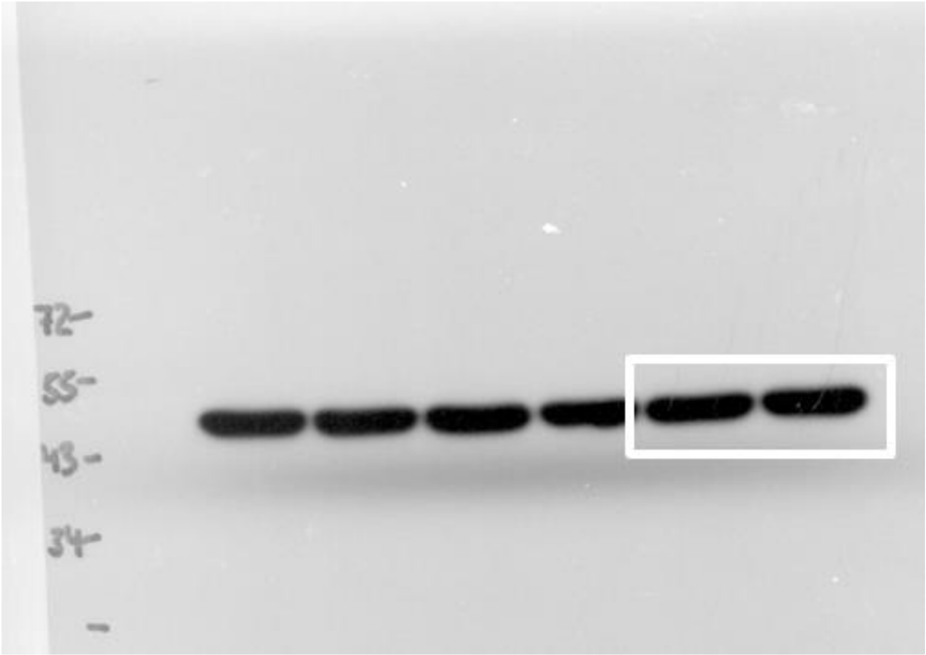

Figure S14. Full-length images of the cropped blots presented in main figure 2D Src transfection (PY-Src, PY-YAP and actin).

PY-Src

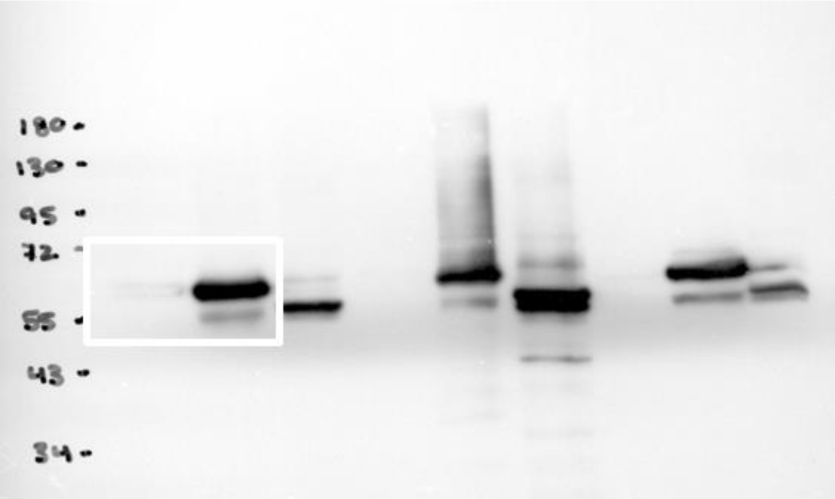

PY-YAP

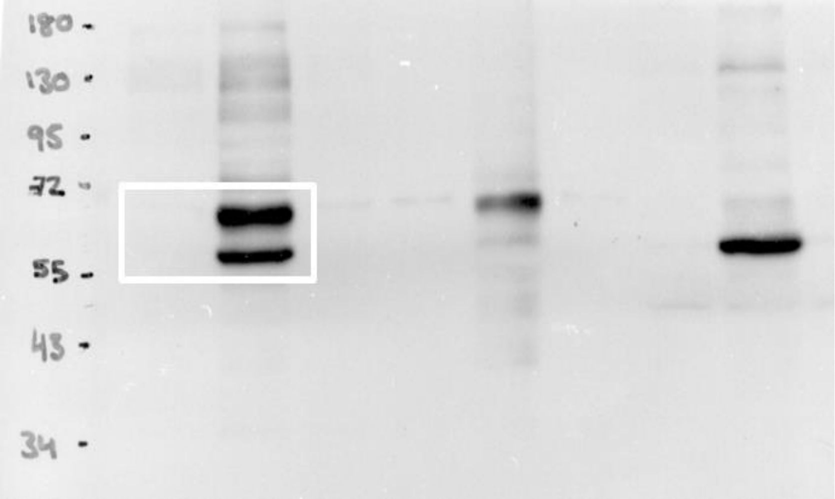

Actin

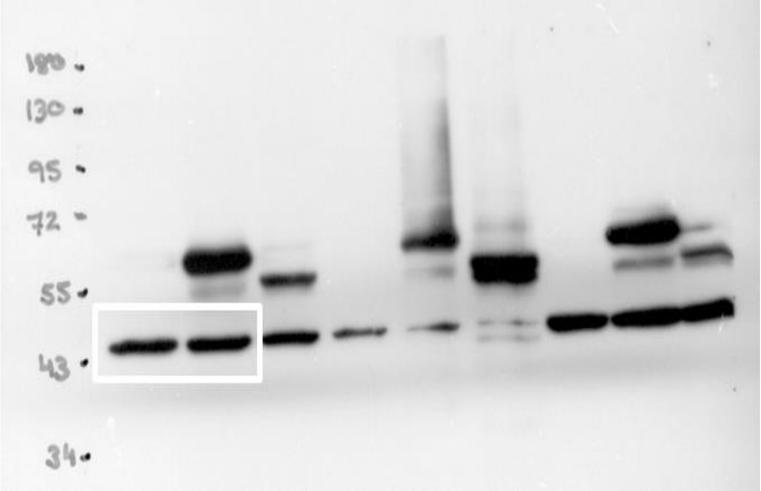

Figure S15. Full-length images of the cropped blots presented in main figure 2D Src transfection (IL6 and actin).

IL6

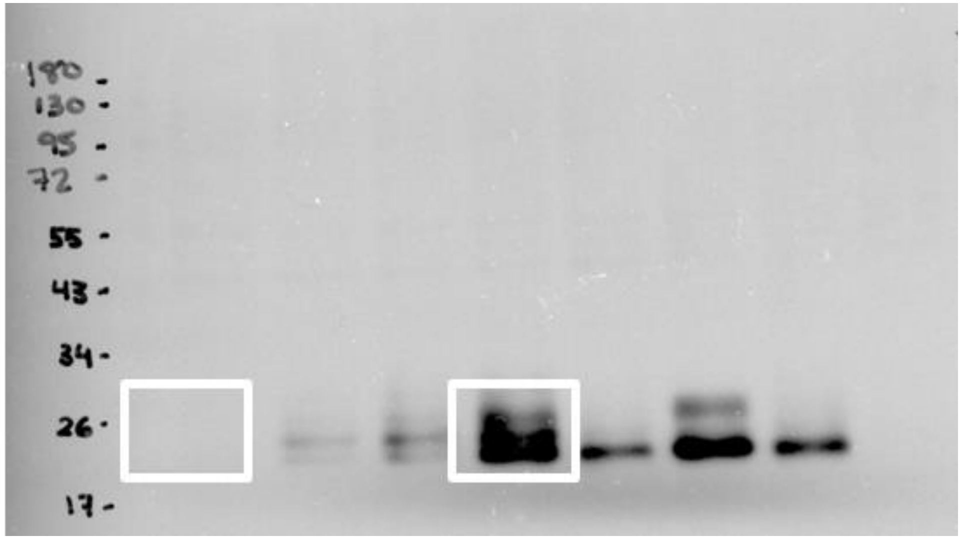

Actin

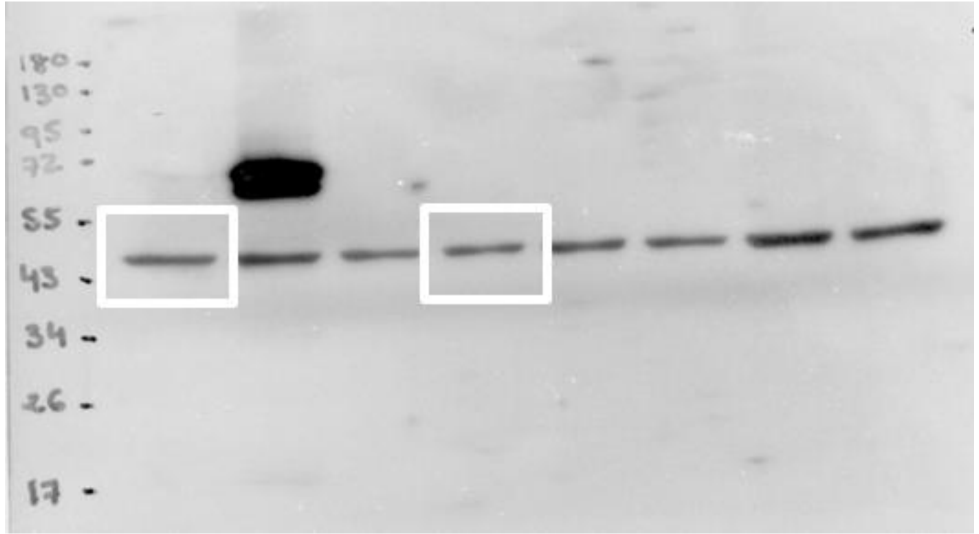

Figure S16. Full-length images of the cropped blots presented in main figure 3E

i21VEGFR1 mRNA

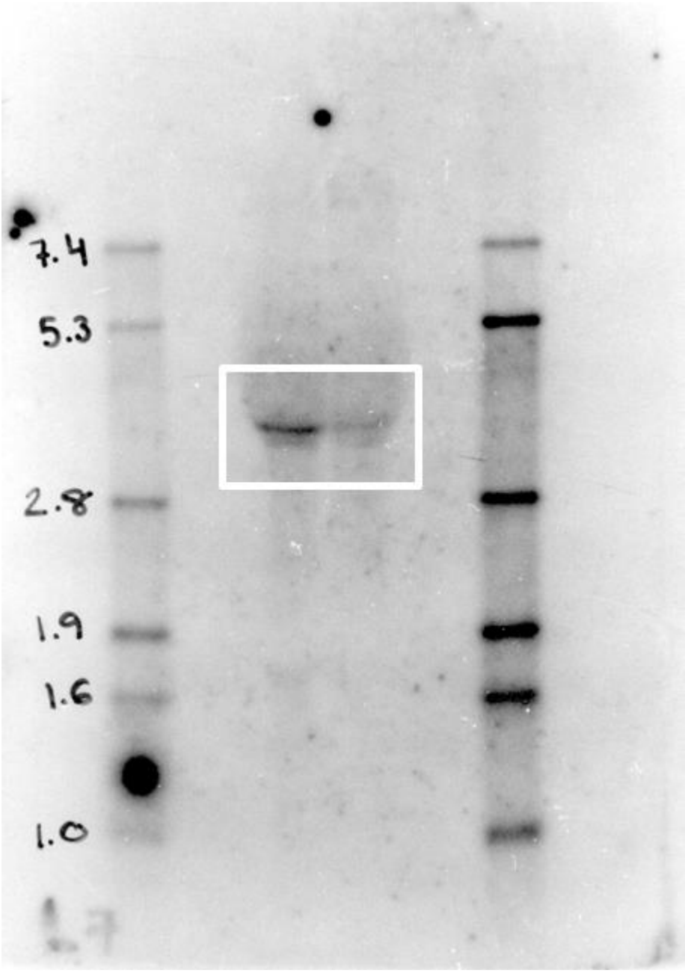

Actin mRNA

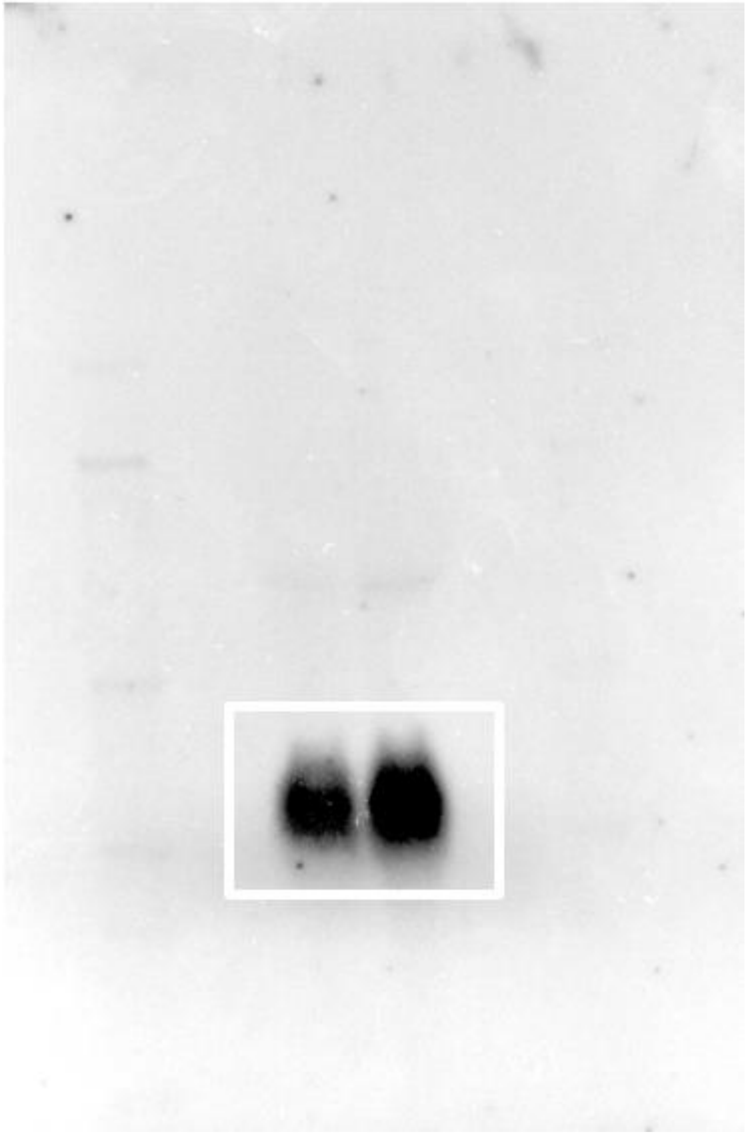

Figure S17. Full-length images of the cropped blots presented in main figure 3E

i21VEGFR1 protein

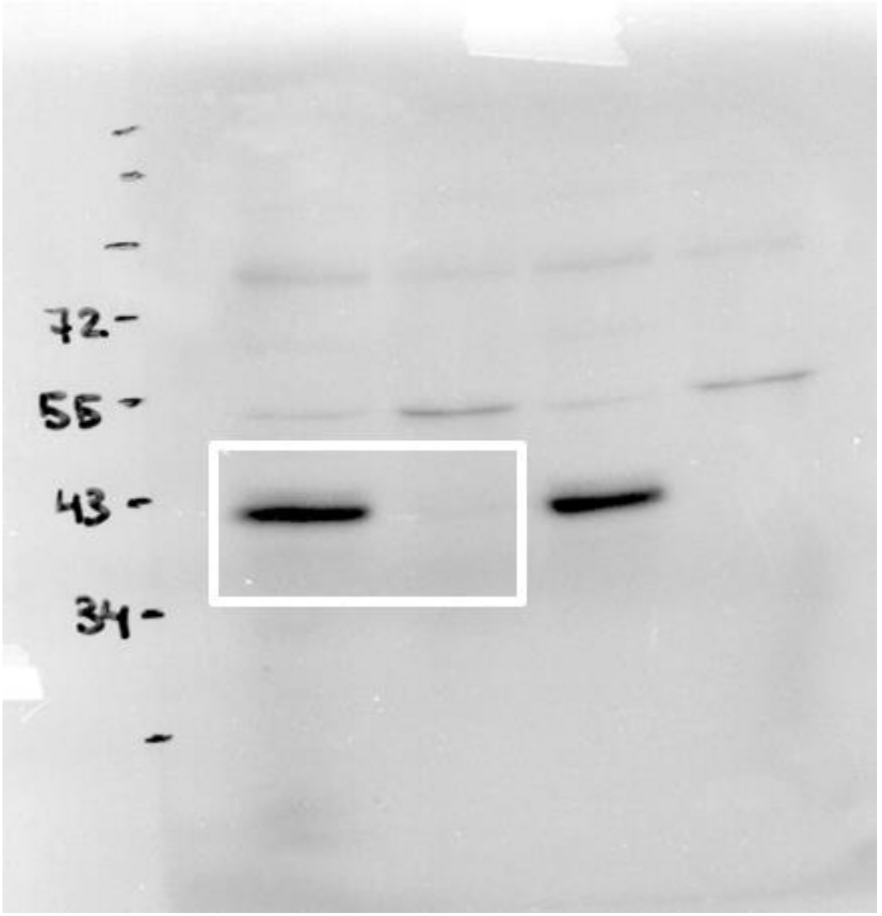

Actin protein

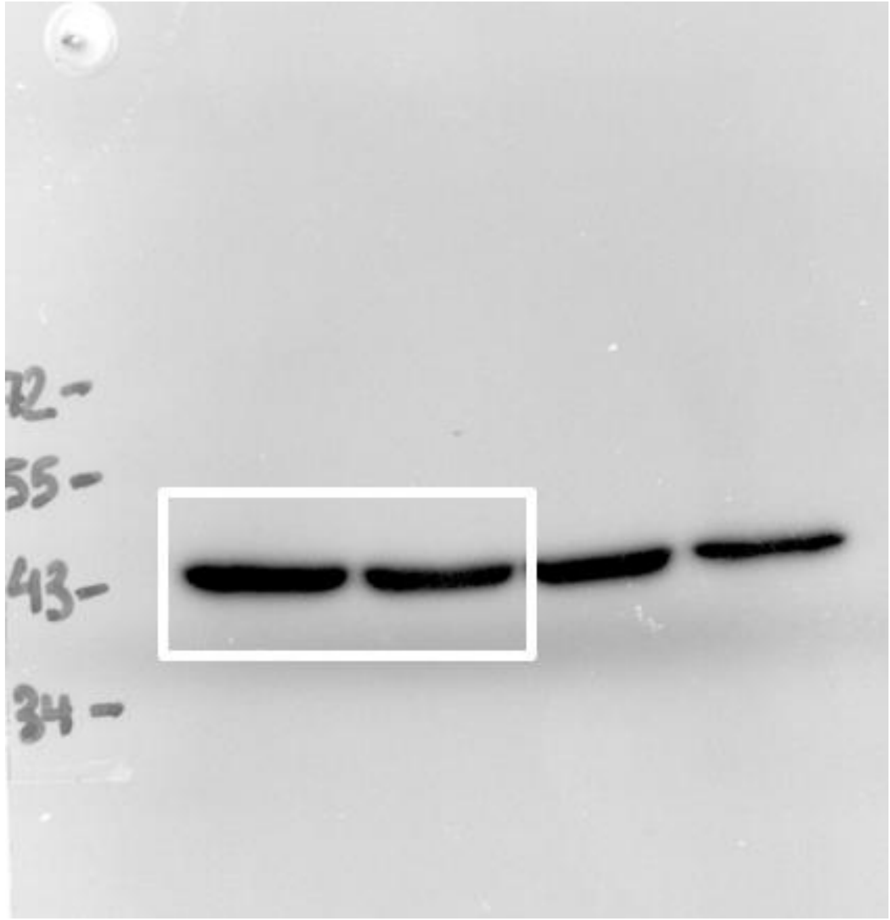

Figure S18. Full-length images of the cropped blots presented in main figure 3E

VEGFR1 mRNA

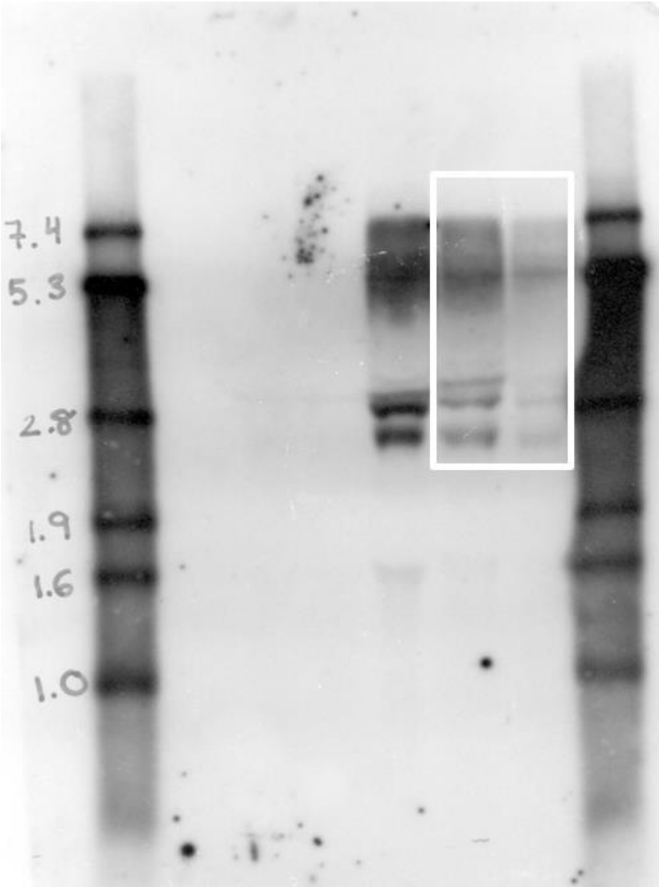

Actin mRNA

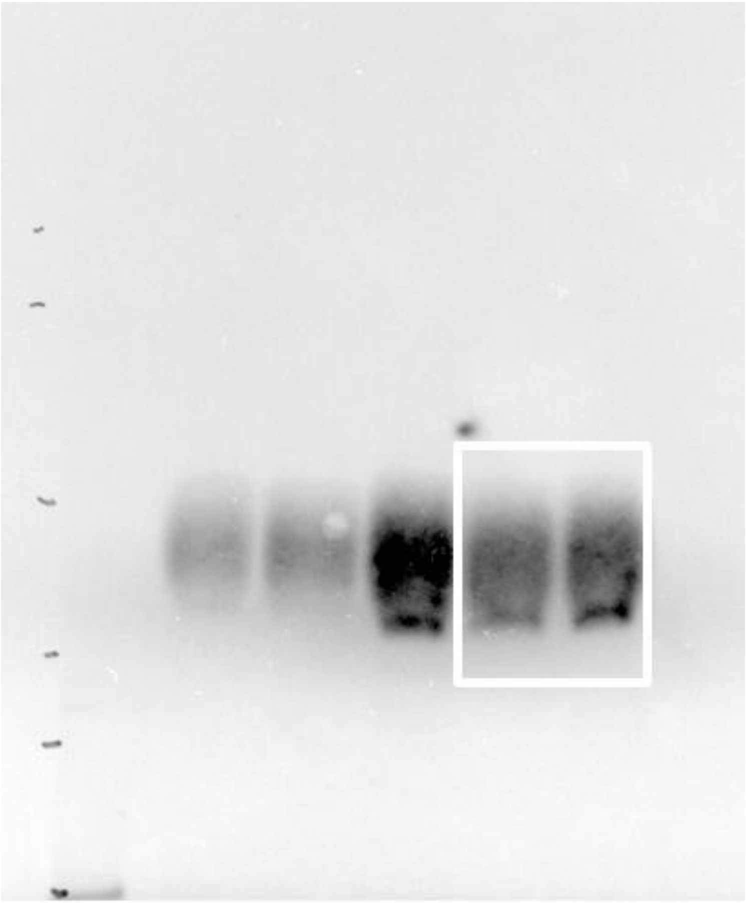

Figure S19. Full-length images of the cropped blots presented in main figure 3E

VEGFR1 proteins

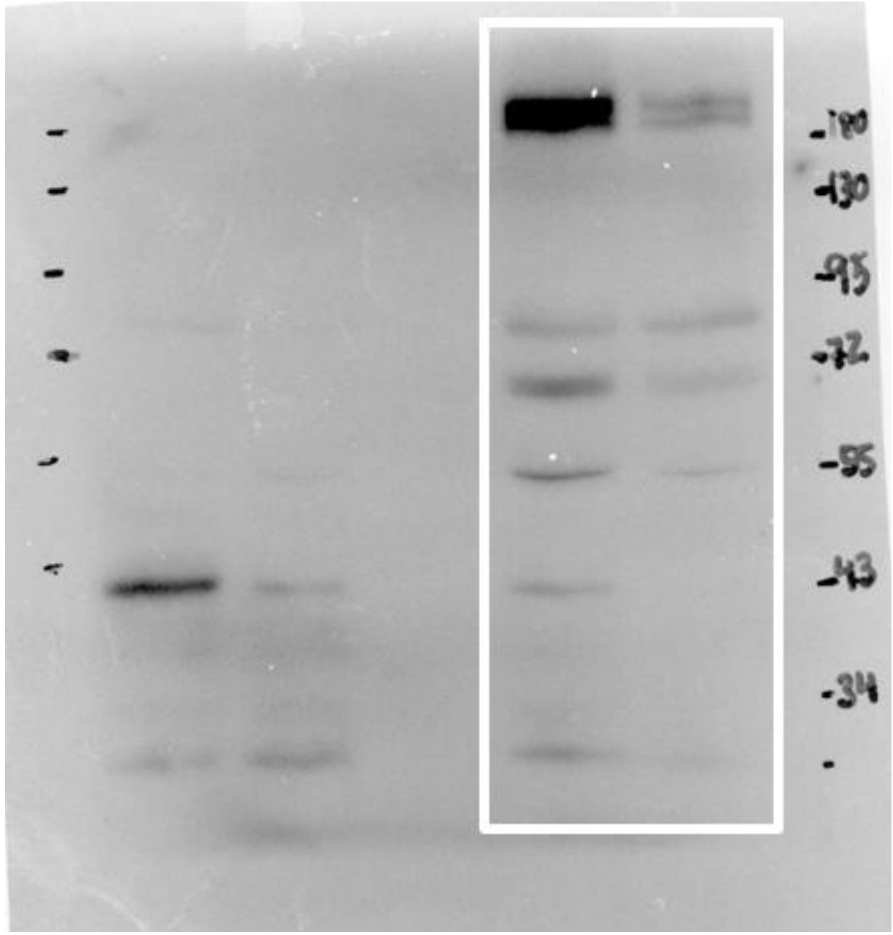

Actin protein

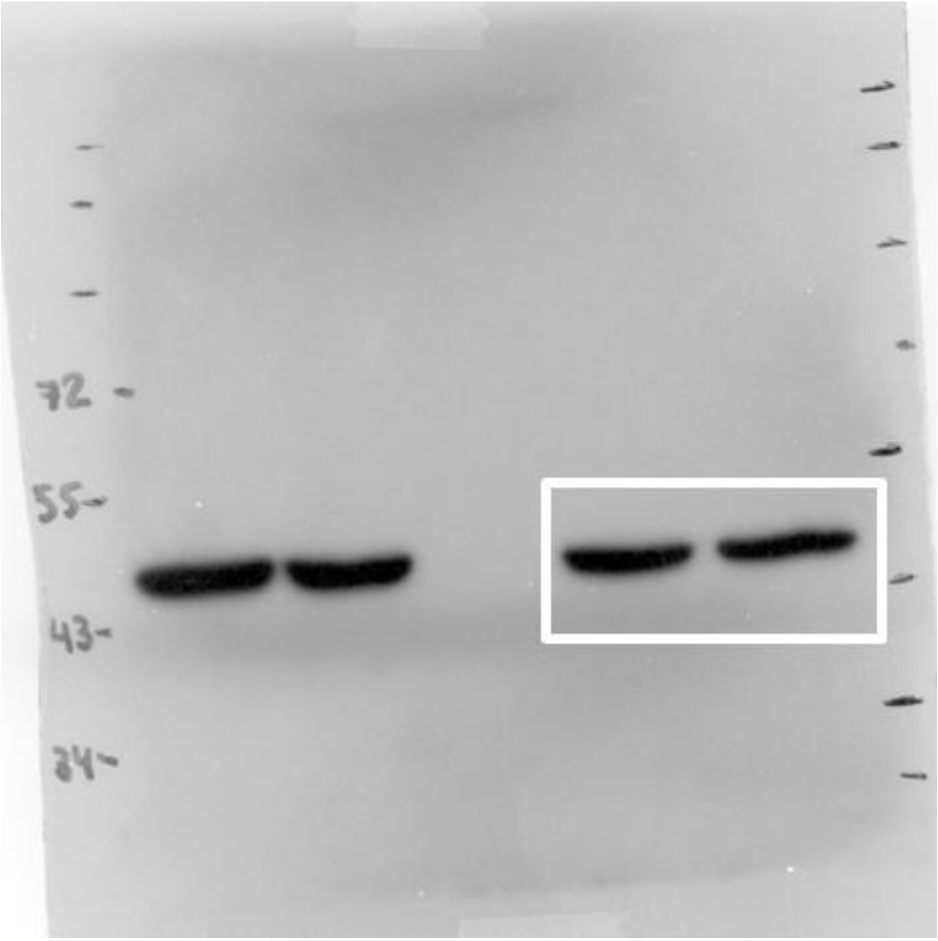

Figure S20. Full-length images of the cropped blots presented in main figure 4A

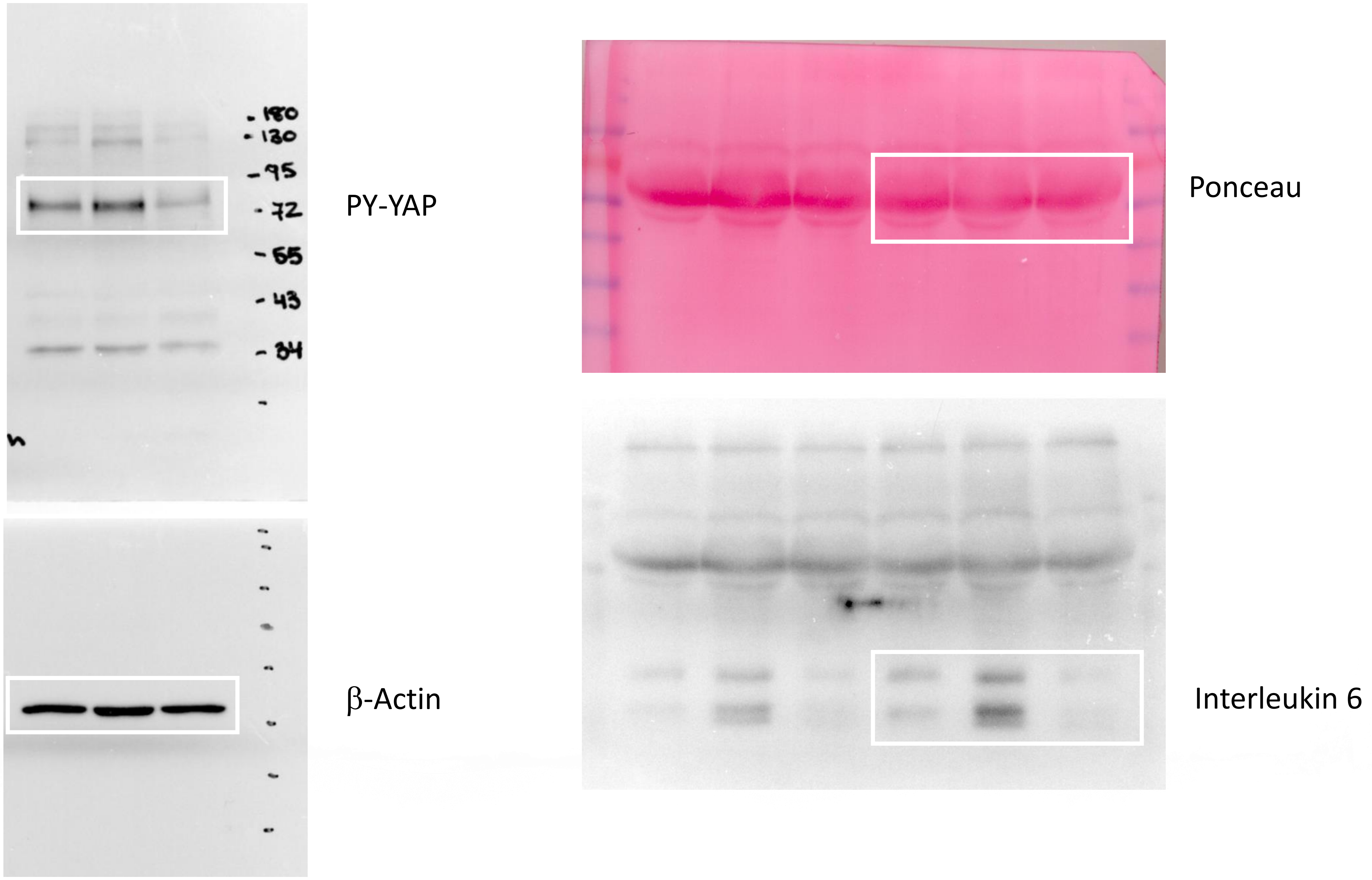

Figure S21. Full-length images of the cropped blots presented in main figure 4B

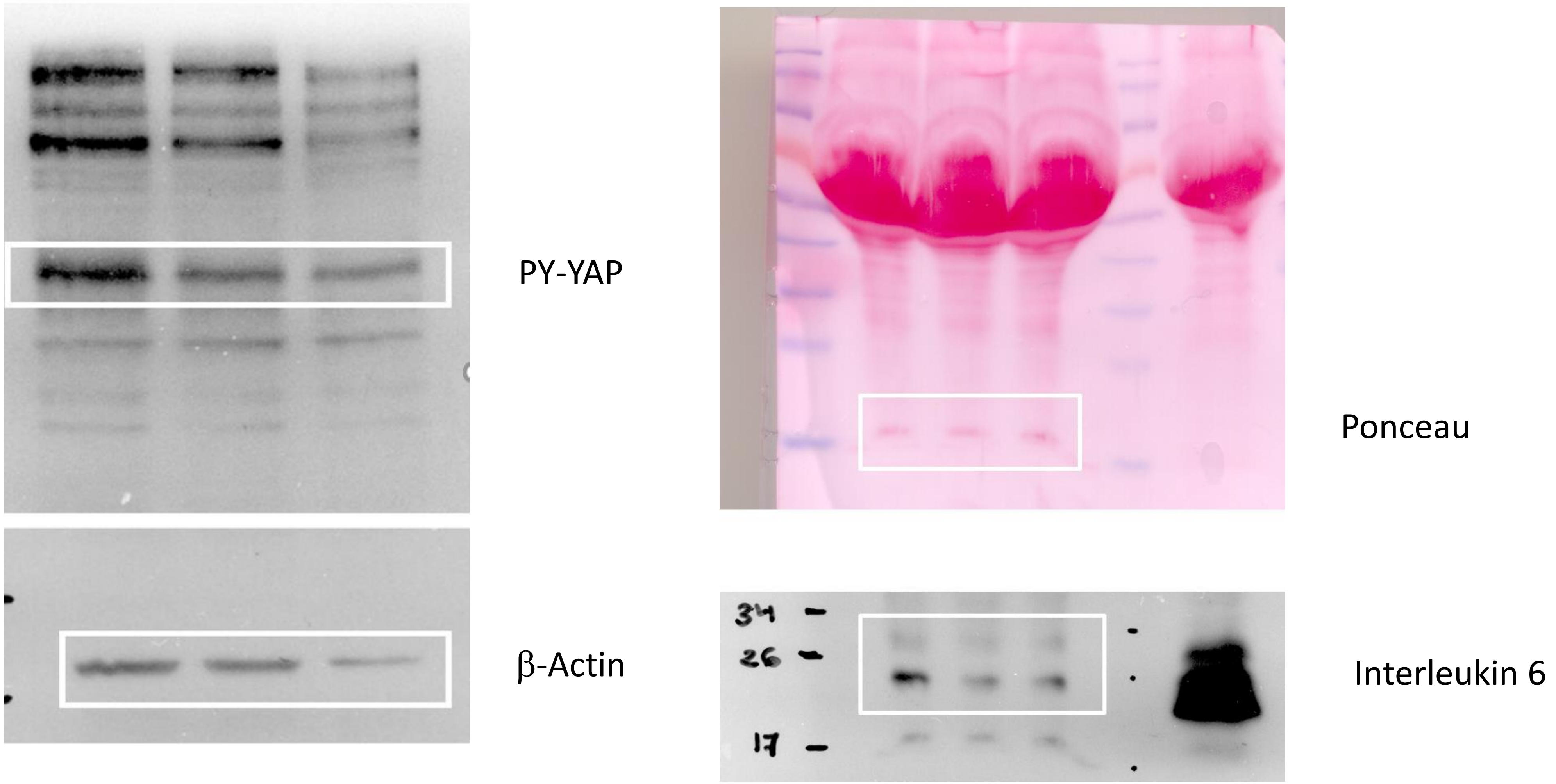

Supplement: Supplementary file 1 — Supplemenary Information [file 41598_2018_25526_MOESM1_ESM.pdf]
